# The Antiquity of the Rhine River: Stratigraphic Coverage of the *Dinotheriensande* (Eppelsheim Formation) of the Mainz Basin (Germany)

**DOI:** 10.1371/journal.pone.0036817

**Published:** 2012-05-16

**Authors:** Madelaine Böhme, Manuela Aiglstorfer, Dieter Uhl, Ottmar Kullmer

**Affiliations:** 1 Department of Geoscience, Eberhard Karls University, Tübingen, Germany; 2 Senckenberg Centre for Human Evolution and Palaeoenvironment (HEP), Tübingen, Germany; 3 Senckenberg Forschungsinstitut, Frankfurt am Main, Germany; Institut de Biologia Evolutiva - Universitat Pompeu Fabra, Spain

## Abstract

**Background:**

Mammalian fossils from the Eppelsheim Formation (*Dinotheriensande*) have been a benchmark for Neogene vertebrate palaeontology since 200 years. Worldwide famous sites like Eppelsheim serve as key localities for biochronologic, palaeobiologic, environmental, and mammal community studies. So far the formation is considered to be of early Late Miocene age (∼9.5 Ma, Vallesian), representing the oldest sediments of the Rhine River. The stratigraphic unity of the formation and of its fossil content was disputed at times, but persists unresolved.

**Principal Findings:**

Here we investigate a new fossil sample from Sprendlingen, composed by over 300 mammalian specimens and silicified wood. The mammals comprise entirely Middle Miocene species, like cervids *Dicrocerus elegans, Paradicrocerus elegantulus*, and deinotheres *Deinotherium bavaricum* and *D. levius*. A stratigraphic evaluation of Miocene Central European deer and deinothere species proof the stratigraphic inhomogenity of the sample, and suggest late Middle Miocene (∼12.5 Ma) reworking of early Middle Miocene (∼15 Ma) sediments. This results agree with taxonomic and palaeoclimatic analysis of plant fossils from above and within the mammalian assemblage. Based on the new fossil sample and published data three biochronologic levels within the *Dinotheriensand* fauna can be differentiated, corresponding to early Middle Miocene (late Orleanian to early Astaracian), late Middle Miocene (late Astaracian), and early Late Miocene (Vallesian) ages.

**Conclusions/Significance:**

This study documents complex faunal mixing of classical *Dinotheriensand* fauna, covering at least six million years, during a time of low subsidence in the Mainz Basin and shifts back the origination of the Rhine River by some five million years. Our results have severe implications for biostratigraphy and palaeobiology of the Middle to Late Miocene. They suggest that turnover events may be obliterated and challenge the proposed ‘supersaturated’ biodiversity, caused by Middle Miocene superstites, of Vallesian ecosystems in Central Europe.

## Introduction

Since the late 18^th^ century fluvial sediments of the Mainz Basin provide exceptionally preserved mammalian fossils, stimulating substantially the early period of vertebrate palaeontology [1], [2]. The earliest find of a fossil primate in 1820 (femur of *Paidopithex rhenanus*, Pohlig 1895) and the description of 19 still valid large mammal species between 1829 and 1834 made localities like Eppelsheim famous worldwide [3]. Because of the common occurrence of deinotheres (Proboscidea) these sediments are known as *Dinotheriensande* [4] and are now defined as Eppelsheim Formation [5], [6], representing the oldest sediments of the Rhine River within the Mainz Basin [7], [6], [8]. It is broadly accepted that the Eppelsheim Formation is of early Late Miocene age (MN9/10, ∼9.5 Ma), based mainly on the presence of typical Vallesian species, such as *Hippotherium primigenium, Tapirus priscus, Aceratherium incisivum, Thalassictis robusta, Macheirodus aphanistus*, *Paramachairodus ogygius*, and others [9], [10], [11], [6].

Here we describe three cervid antlers, deinothere tooth material, and a silicified wood discovered in the early 1980ies in the locality Sprendlingen ( = Steinberg, Napoleonshöhe, [12]), directly beneath a layer containing the only known fossil macroflora from the Eppelsheim Formation [13]. We evaluate biostratigraphic significance of Miocene deer, silicified wood, and leaf flora and analyze the latter palaeoclimatically. Our results indicate a Middle Miocene age of this Sprendlingen sample, which raises questions about stratigraphic coverage of the Eppelsheim Formation, and may have important consequences for Miocene cervid diversity, mammalian biostratigraphy, metacommunity studies, and basin evolution including the age of the Rhine River.

### Geological Setting

**Figure 1:**
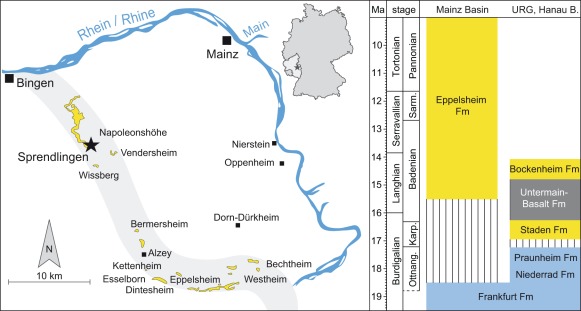
Geography and Stratigraphy. Left: Geographic position of the Sprendlingen locality. Outcrops of the Eppelsheim Formation are displayed in yellow and the reconstructed course of the proto-Rhine is marked in grey (according to Franzen [102]). Right: Lithostratigraphy of Mainz Basin, Upper Rhine Graben (URG) and Hanau Basin and their chronostratigraphic correlation (international and regional stages) according to [98], [14]. Eppelsheim Formation is modified according present results. Coloring represent carbonatic (blue) and siliciclastic (yellow) depositional systems. Abbreviations: Ottnan.  =  Ottnangian, Karp.  =  Karpatian, Sarm.  =  Sarmatian.

The Sprendlingen sandpits are situated 3 km northeast of Sprendlingen (22 km southwest of Mainz), 250 meters above sea-level on the elevation Steinberg (Napoleonshöhe) in the western part of the Mainz Basin (Fig. 1). The fossil-bearing section has been described by Meller [13] and is here named Sprendlingen 2 (coordinates N 49° 53′04′′, E 8° 00′ 37′′). It is now covered by rubbish dump. Active outcrops show basically the same sedimentary features 500 meters further east, though. According to Meller [13] and our investigations in the field the siliciclastic succession (Eppelsheim Formation) rests with erosional contact on marls of the Frankfurt Formation [14], rich in minute *Hydrobia* shells. The upper 25 cm comprise olive-brown, decalcified clay, containing numerous septarian limestone nodules. The base of the Eppelsheim Formation is a 45 cm thick coarse sand to medium-grained gravel with intense impregnation of ferric and manganese oxides. This layer contains all mammalian fossils, as well as the silicified wood. According to observations by Meller [13] up-section follow unconformably 25 cm medium-grained ferruginous gravels containing reworked clays, overlain by about 80 cm of grey to yellowish clays. Especially in its middle part the latter contain a rich leaf and seed flora described by Meller [13]. This clay horizon seems to be laterally eroded, and is not preserved in todaýs outcrop. The upper ∼8 m of the latter is composed of yellowish to whitish cross-bedded medium to coarse sands with rare fine-grained gravel layers intercalated. It contains layers of reworked clays and silts, as well as limonitic-filled holes of oxidized tree-trunks.

## Results

### Systematic Palaeontology

Class Mammalia Linnaeus 1758

Order Artiodactyla Owen 1848

Suborder Ruminantia Scopoli 1777

Infraorder Pecora Linnaeus 1758

Family Cervidae Goldfuss 1820

Genus *Dicrocerus* Lartet 1837

Type species: *Dicrocerus elegans* Lartet 1837

Valid species: *Dicrocerus elegans* Lartet 1837

***Dicrocerus elegans*** Lartet 1837

Holotype: not designated

Type locality: Sansan, France

Stratigraphic range: early Middle Miocene, Langhian to Early Serravallian

Material: SSN 12SP2, NHMM P3712

#### Description

**Figure 2:**
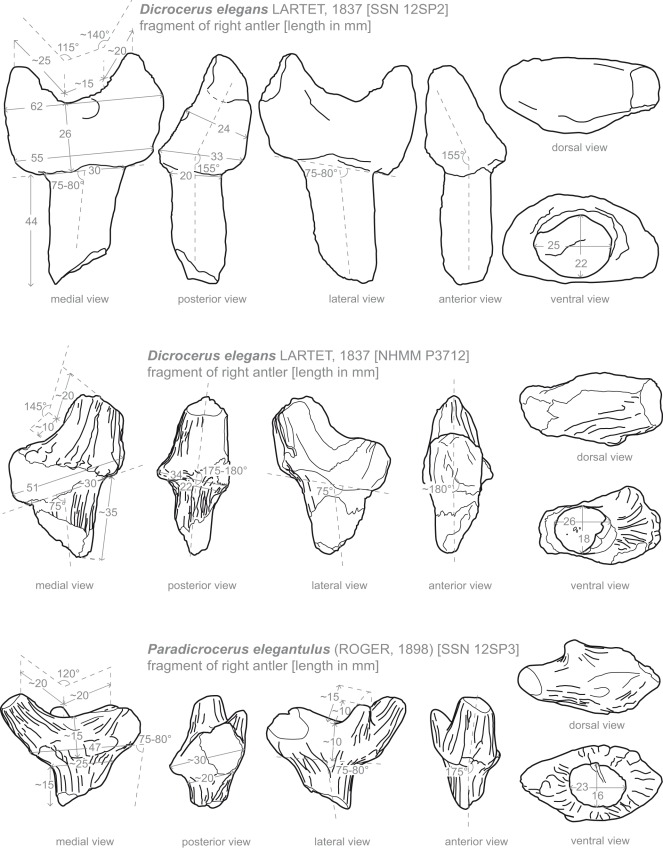
Drawings of cervid antlers from Sprendlingen 2 with dimensions measured.

**Figure 3:**
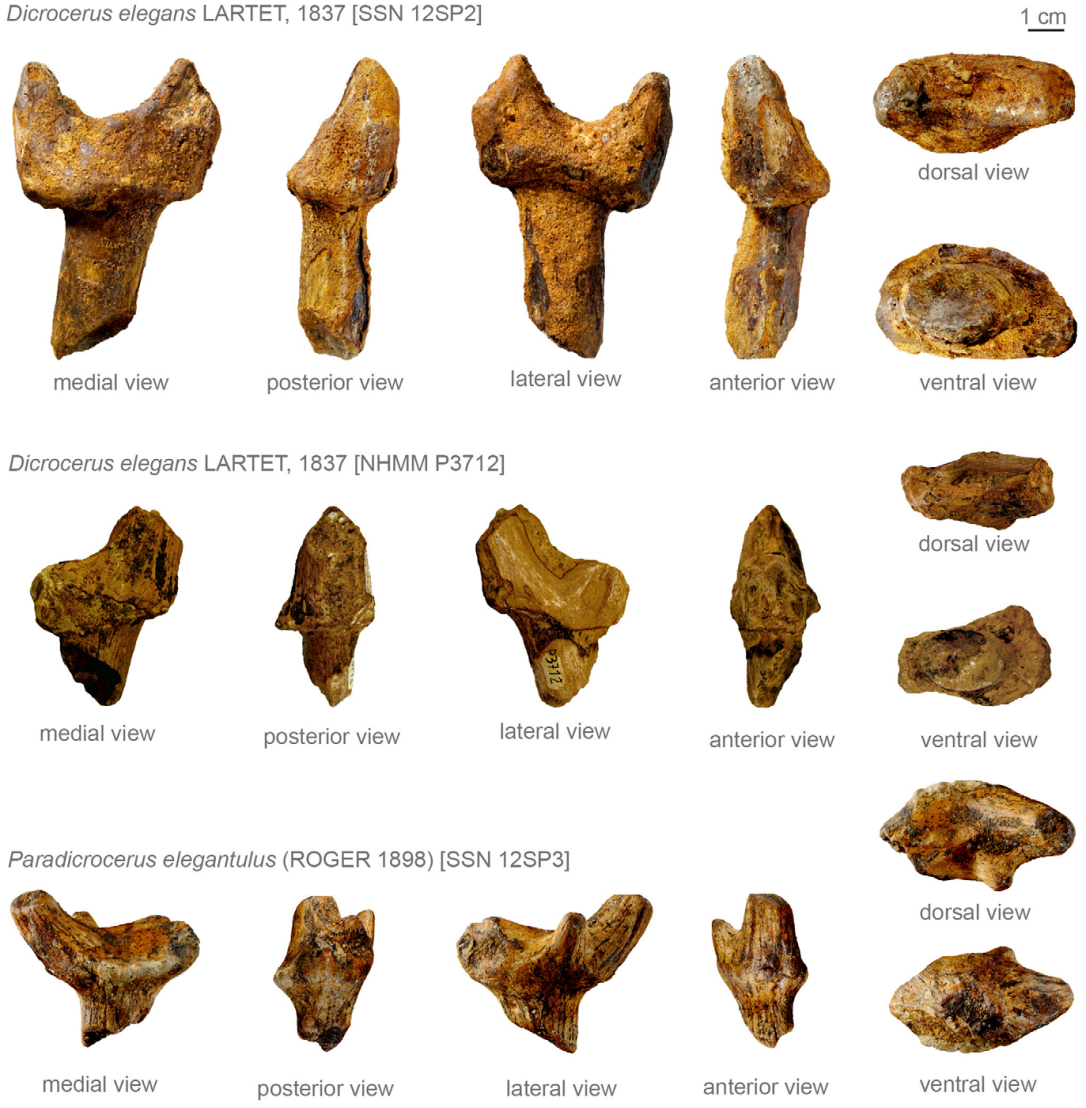
Photo images of antlers from Sprendlingen 2. Photos of SSN 12SP2 and 12SP3 by W. Gerber, photo of NHMM P3712 by M. Aiglstorfer (both Eberhard Karls Universität Tübingen).

SSN 12SP2 is a right cervid protoantler showing the distal part of the pedicle and a protoburr with two prongs, missing most of the posterior appendage (Figs. 2, 3). The at least 44 mm long pedicle ends distally in a protoburr, clearly separated from the pedicle by an abrupt increase in diameter. Proximally the pedicle has an anteroposterior elongated sub-ellipsoid cross section, evolving distally more oval. The protoantler does not show a modern burr with a clearly defined corona of pearls but a protoburr with a weak sculptured outline. There is no constriction of the protoantler above the basal plate. The plate itself has a sub-ellipsoid shape with a flattened lateral side. Two simple prongs, the anterior being the shorter one, arise about 25 mm above the basis of the basal plate separated by a 15 mm long inter-space. An additional small elevation is visible at the median side of the base, being less than 10 mm in diameter, and only a few millimeters high. The prongs are inclined medially and laterally enclose an angle of about 155° with the pedicle in anterior and posterior view, giving the protoantler a lateral expansion. The anterior inclination of the basal plate with the pedicle is less with an approximate angle of 75° to 80°. Due to intensive incrustation with iron hydroxide it cannot be cleared, whether the anterior appendage was longer originally or was broken off. The incrustation furthermore does not allow any conclusions on the ornamentation.

NHMM P3712 is also a right protoantler (Figs. 2, 3), but more fragmentarily preserved than specimen SSN 12SP2 and comprises the distal part of the pedicle, a strongly fragmented protoburr and two very fragmentary prongs. Only a short part of the anterior prong is preserved. The posterior prong was probably larger. Of its length 20 mm are preserved. It shows a sub-triangular cross section. While the transition area pedicle/basal plate is clearly stepped, the posterior prong arises without distinct constriction out of the basal plate, which forms a protoburr. A corona like structure surrounding the basal plate can be reconstructed. The basal plate itself is preserved too fragmentarily to allow a reconstruction of its shape. It is inclined anteriorly and encloses an angle of about 75° with the pedicle. The pedicle shows an ellipsoid anteroposterior elongated cross section. The posterior prong does not show a strong medial inclination in posterior view and encloses an angle of about 175–180° with the pedicle, indicating an only minor lateral expansion. Although poorly preserved and incrusted with iron hydroxide, some of the protoantler’s surface ornamentation composed of intense longitudinal ridges and grooves on the posterior prong and weaker longitudinal lines along the pedicle can still be observed.

#### Comparison

According to size and morphology (Figs. 2, 3) SSN 12SP2, NHMM P3712 should be assigned to *Dicrocerus elegans*. Both protoantlers show a clear separation of the pedicle from the protoburr, a dichotomy with a longer posterior prong, a laterally inclined basal plate (strongly in SSN 12SP2; only weak in NHMM P3712), and medially inclined prongs (clearly in SSN 12SP2), giving the antler a lateral expansion like in *Dicrocerus elegans* [15], [16], [17], [18], [19], [20], [21]. Furthermore the specimens have in common with *D. elegans* the long oval cross section of the pedicle [15], [16], the derivation of the prongs immediately out of the protoburr [15], [17], [19] and the presence of an interspace between the two prongs [16], [17], [19]. Measurements of the specimens are in the range of the *D. elegans*, as well [16], [17], [19].

Protoantlers SSN 12SP2, and NHMM P3712 clearly differ from similar sized cranial appendages like *Paradicrocerus elegantulus* (Roger 1898) by a clear dichotomy [22]. Furthermore specimen SSN12SP2 clearly shows a less developed lateral expansion of the basal plate proportionally to the protoantler’s expansion (indicated as well in the fragmentarily preserved NHMM P3712), than observed in *Paradicrocerus elegantulus* by [19]. Miocene cervids with dichotomous protoantlers/antlers like *Acteocemas* Ginsburg 1984, *Procervulus* Gaudry 1877, *Heteroprox* Stehlin 1928 and *Euprox* Stehlin 1928 differ from the specimens by smaller dimensions and a more delicate habitus [23], [24], [18], [25], [20]. The basal plate in *Euprox* from Steinheim shows an average length of about 30 mm (pers. obs.), while it measures more than 50 mm in length in the here described specimens. The distinct separation of the basal plate from the pedicle forming a protoburr clearly distinguishes SSN 12SP2, and NHMM P3712 from *Procervulus*, lacking a burr generally [24], [25], and *Acteocemas*, and *Heteroprox*, normally missing a pronounced basal plate or a clearly defined burr [26], [17], [18], [25], [19], [20]. In contrast to *Euprox* the specimens have a burr without pearls and distinct constriction above the basal plate [17], [25], but show a derivation of prongs immediately out of a protoburr. Furthermore the basal plate in *Euprox* is less expanded [27], [17], [25]. In contrast to *Euprox* and *Heteroprox* from Steinheim (pers. obs.) the prongs of specimen SSN 12SP2 form a wider angle with the pedicle in anterior view giving the protoantler a larger lateral expansion. In contrast to the here described specimens the pedicle in *Amphiprox anocerus* (Kaup 1833) is widening gradually before ending in a burr [17]. Furthermore the dichotomous furcation is higher above the burr in the latter [17].

Genus *Paradicrocerus* Gabunia 1959

Type species: *Paradicrocerus flerovi* Gabunia 1959

Valid species: *Paradicrocerus flerovi* Gabunia 1959, *Paradicrocerus elegantulus* (Roger 1898)

***Paradicrocerus elegantulus*** (Roger 1898)

Holotype: frontal with fragmentary antlers (NMA 795004)

Type locality: Stätzling, Germany

Stratigraphic range: early Middle Miocene, Langhian

Material: SSN 12SP3

#### Description

Specimen SSN 12SP3 is a right protoantler, with abrupt pedicle and prongs (Figs. 2, 3). The transitional area pedicle/basal plate shows a gradual expansion. It is not stepped. At least three irregularly prongs arise from the basal plate. The pedicle shows an elongated ellipsoid, the basal plate a rounded lanceolate cross section. The latter is only little inclined medially to the longitudinal axis of the pedicle. A protocoronet is developed as a distinct collar at the median side, comprising several small elevations, whereas on the lateral side the ornamentation is quite weak. The posterior appendage has the largest base and erupts directly out of the protocoronet. Supposedly it comprises two or more prongs evolving very close to each other and splitting more distally. The largest definitely single branch is the anterior one. Its cross section is sub-triangular to sub-rounded. The base of the bifurcation of the two main branches is about 15 mm above the base of the collar like structure. Laterally a third much smaller prong erupts about 10 mm above the protocoronet. As it is broken off rather distally an original length of roughly 15 mm could be estimated. The basal plate forms a wide and shallow plane. Its dorsal surface shows rests of ridge-like structures sub-parallel to its longitudinal axis. Most of the protoantler’s expansion originates from the large size of the antler base, while the prongs contribute only little to it. With an angle of 175° enclosed by the pedicle and the anterior prong no lateral expansion is caused by medial inclination of the prongs. Although abraded, the protoantler’s surface still shows an ornamentation consisting of weak longitudinal lines.

#### Comparison

The specimen shows great affinities to holo- and paratypes of *Paradicrocerus elegantulus* from Stätzling [28], [22]. With this species it has in common the rounded lanceolate cross section of the basal plate, the extension of the protoantler mainly by the basal plate [29], [28], [22], the gradually widening of the pedicle ending in the protoantler base and the possession of more than two dominant prongs [29], [28], [22]. Furthermore general dimensions and habitus of SSN 12SP3 are in the range of the type series (NMA 795004 (holotype), NMA 795047, NMA 795041-3, NMA 795046; best consistency with holotype). Especially the shallow but wide basal plate with ridges covering the dorsal surface fit well to observations on the type series, distinguishing specimen SSN 12SP3 furthermore from other Miocene cervids, not showing this feature in burr/protoburr. A protocoronet, as observed in *Paradicrocerus elegantulus* [30], is still recognizable in the specimen even though its surface is slightly abraded. Like in *Dicrocerus* and *Paradicrocerus* the prongs erupt directly out of the basal plate in the specimen and the distal pedicle is laterally compressed [15].

With more than two distinct prongs the antler clearly differs from Miocene cervids with dichotomous protoantlers and antlers like *Acteocemas*, *Dicrocerus*, *Procervulus*, *Heteroprox*, and *Euprox* [16], [17], [24], [18], [25], [19], [20], [21]. Furthermore the furcation and length of the prongs contributes more to the extension of the antler in these species than does the basal plate [16], [17], [24], [18], [25], [19], [21], whereas it is vice versa in the here described specimen, as mentioned above. The gradual widening at the distal part of the pedicle resulting in the basal plate distinguishes the antler furthermore from *Euprox* and *Dicrocerus* both showing a stepped transition area pedicle/burr [16], [17], [25], [19], [20]. The rounded, lanceolate cross section of the basal plate differs from *Dicrocerus* [16], [17], which has a rounded to ellipsoid basal plate shape. In contrast to *Heteroprox*, *Procervulus,* and lagomerycids [15], [17], [24], [25], [19], [20] the specimen possesses a protocoronet.

Order Proboscidea Illiger 1811

Family Deinotheriidae Bonaparte 1845

Genus *Deinotherium* Kaup 1829

Type species: *Deinotherium giganteum* Kaup 1829

Valid species: *Deinotherium giganteum* Kaup 1829, *D. cuvieri* Kaup 1832, *D. bavaricum* von Meyer 1831, *D. levius* Jourdan 1861, *D. gigantissimum* Stefanescu 1892 ( =  *D. pravum* Eichwald 1835)

***Deinotherium bavaricum*** von Meyer 1831

Lectotype: right p3 (BSPG AS I 220)

Type locality: Georgensgmünd, Bavaria, Germany

Stratigraphic range: early Middle Miocene, Langhian to Early Serravallian

Material: SSN 12SP 4–11, 13, 14, 16–21, 24, 28–30, 35–39; NHMM P 3688–3692, 3696, 3697, 3700, 3736, 3742, 3863, 3865–3870, 3872, 3876, 3877, 3881, 3886, 3888, 3892, 3895, 3995.

***Deinotherium levius*** Jourdan 1861

Lectotype: right maxilla (Natural Science Museum, Lyon, ML L.Gr. 962)

Type locality: La Grive Saint-Alban M, Isère, France

Stratigraphic range: late Middle Miocene, Late Serravallian

Material: SSN12SP 12, 15, 22, 23, 25–27, 31–34; NHMM P 3698, 3699, 3875, 3889.

Tooth morphology of different deinotheres is quite similar and species are mainly distinguished by size [31], [32], [33], [34], [35]. We compare the Sprendlingen 2 deinotheres with well described and rich samples from three different time periods, comprising three species (following the taxonomic concept of Ginsburg and Chevrier [34]). For *Deinotherium bavaricum* we choose the sample from the Falun de la Touraine and Anjou (early Middle Miocene marine sediments representing the Langhian transgression; 15±0.5 Ma; assigned to mammal ‘zone’ MN 5), described by Ginsburg and Chevrier [34]. A representative sample of *D. giganteum* comes from Montredon (a late Vallesian MN 10 site from Herauld, France; ∼9.5 Ma), described by Tobien [36]. Stratigraphic intermediate comparative samples comprise specimens either described as *D. levius* (late Middle Miocene MN 8 sites from France and Germany: La Grive, St. Gaudens, Tournan, Massenhausen, Hinterauerbach; 13 to 11.5 Ma; [32], [34]) or as *D. giganteum* (early Late Miocene/early Vallesian MN 9 sites from Germany and Austria; 11.5 to ∼10 Ma; [32], [35]), including the type material from Eppelsheim described by Kaup [37].

**Figure 4:**
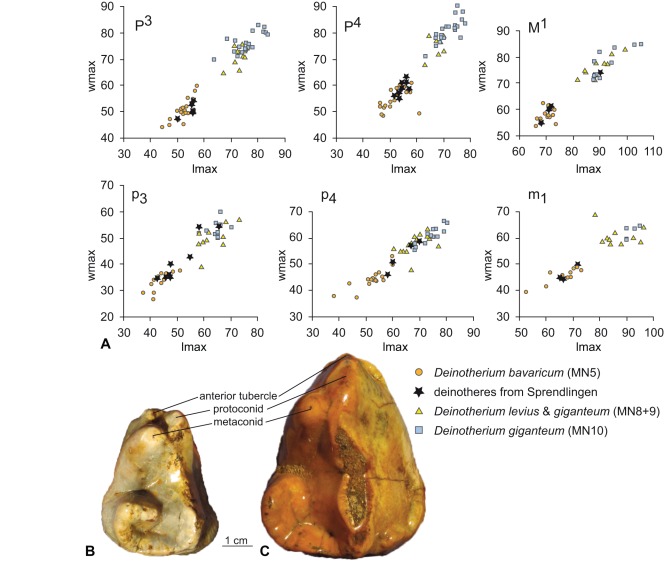
Deinothere tooth metrics and morphology of p3. A: Length–width diagram of upper and lower P3-M1 molars of *Deinotherium bavaricum*, *D. levius, D. giganteum* (data mainly from [32], [36], [34], [35]; for full reference and data set see supplementary Table S1). B: p3 of *Deinotherium bavaricum* (Sprendlingen 2, SSN 12 SP30). C: p3 of *Deinotherium levius* (Sprendlingen 2, SSN 12 SP31). Photos by M. Böhme and M. Aiglstorfer.

The majority of the Sprendlingen 2 teeth (51 out of 66), representing all permanent tooth positions, fall within the range of the small-sized species *Deinotherium bavaricum* from the early Middle Miocene (Fig. 4). Sizes of P4, p3, p4, and m1 correspond best to the upper range of the Falun sample. However, 15 teeth (D3, M1, M2, M3, p3, p4, and m2) are significantly larger and fall within variation of the larger sized deinothere species *D. levius* and *D. giganteum*. According to Gräf [32] *Deinotherium levius* and *D. giganteum* are metrically similar, but can be differentiated by morphologic characters on p3 [32], [38], contra [35]: 244). In the p3 of *D. levius* metaconid and protoconid are separated like in *D. bavaricum* (Fig. 4), whereas they are fused in *D. giganteum*. The metaconid is shifted slightly posterior and the anterior tubercle is reduced in *D. levius* in comparison to *D. bavaricum* [32], [38], [39]. In the Sprendlingen 2 material all large sized p3 (SSN12SP 31–33) show well separated anterior conids (Fig. 4), a metaconid slightly shifted posterior, and a reduced anterior tubercle. These characters clearly distinguish the specimens from the same sized teeth of *D. giganteum*, and the smaller sized teeth of *D. bavaricum* (Fig. 4). We therefore conclude that beside the small-sized *Deinotherium bavaricum* a second, large-sized species is present in Sprendlingen 2, which should be referred to *Deinotherium levius*, based on the morphology of the p3.

### Taphonomy

With iron-hydroxide incrustation also of aborted parts of pedicle and prongs, as well as signs of abrasion the protoantlers (especially SSN 12SP2, and NHMM P3712) show indications for redeposition. In his revision of the *Dinotheriensand* cervids Haupt [17] observed that antlers of *Amphiprox anocerus* show only little abrasion, whereas those of *Euprox furcatus* and especially *Heteroprox larteti* and *Dicrocerus elegans* exhibit the most intense evidence for physical reworking.

Sprendlingen 2 deinotherid teeth, regardless their taxonomic attribution, show only minor signs of physical alteration on enamel cusps, although roots are commonly abraded or broken.

### The Stratigraphy of Miocene Deer in Central Europe

To evaluate stratigraphic distribution of both Sprendlingen 2 deers, *Dicrocerus elegans* and *Paradicrocerus elegantulus,* we investigate the stratigraphy of the family Cervidae (excluding *Lagomeryx*) in Central Europe during the Miocene. This stratigraphic up-date is based on recent advances in Central European chronostratigraphy and mammal biostratigraphy (e.g.: [40], [41], [42], [43], [44], [45], [46], [47], [48]. In total we explored 66 cervid-bearing localities mainly from the North Alpine Foreland Basin and the Central Paratethyan Vienna and Styrian Basins (excluding localities from the *Dinotheriensande*), spanning 9 million years from 18.5 to 9.5 Ma. This comprehensive database provides a far more detailed insight into chronology of Central European cervids, well beyond the resolution of the MN-‘zonation’.

**Figure 5:**
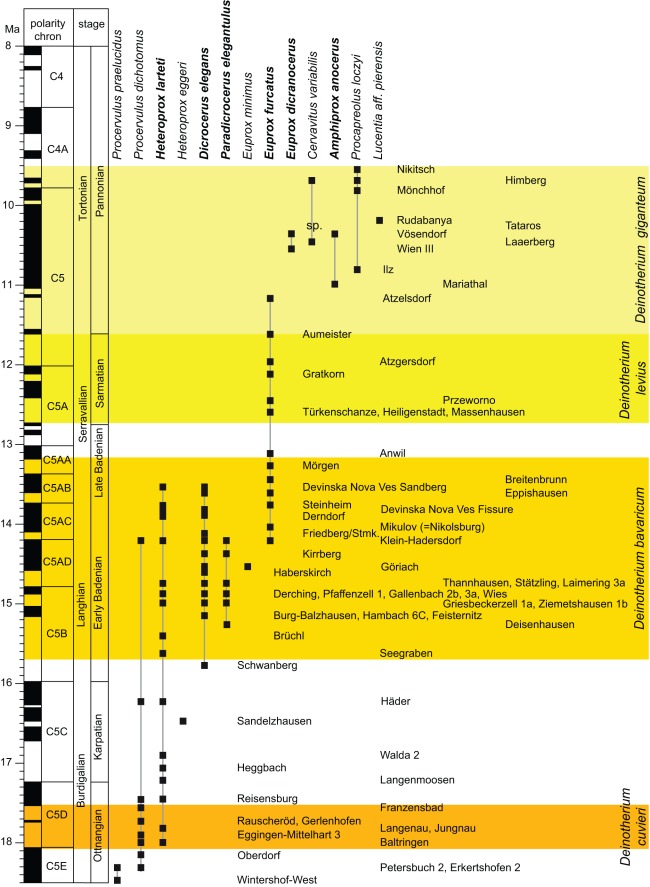
Stratigraphic distribution of Miocene Cervidae (excluding *Lagomeryx*) in Central Europe. Cervid species documented from the Eppelsheim Formation [17] are shown in bold. Yellow to orange shaded areas display stratigraphic distribution of *Deinotherium* species. Reference to all localities can be found in supplementary Table S2.

During the investigated time-period 13 non-*Lagomeryx* cervid species are known from Central Europe (Fig. 5). The late Early Miocene (late Burdigalian; Ottnangian and Karpatian) is characterized by four species of the genera *Procervulus* (*P. praelucidens, P. dichotomus*) and *Heteroprox* (*H. larteti, H. eggeri*). At the beginning of the Middle Miocene (Langhian; Early Badenian) *Dicrocerus elegans* immigrates, soon followed by *Paradicrocerus elegantulus*, both coexisting with *H. larteti*. The rare species *Euprox minutus* appears in the late Early Badenian. Around the Middle Badenian (or the Early-Late Badenian transition) *Paradicrocerus* and possibly the last representative of *Procervulus* disappear, whereas *H. larteti* and *D. elegans* still coexist during the earlier part of the Late Badenian. Contemporary with the last occurrence of *Paradicrocerus* and *Procervulus* the first representatives of *Euprox furcatus* immigrate. This species represents the only deer in Central Europe from the late Late Badenian until the beginning of the Late Miocene (Tortonian, Pannonian). In the early Pannonian a new turnover event occurs, with the replacement of *E. furcatus* by several immigrants or syntopically evolving species like *E. dicranocerus, Amphiprox anacerus, Cervavitus variabilis, Procapreolus loczyi*, and *Lucentia* aff. *pierensis*. Stratigraphically younger record is not investigated here, but literature data suggest low cervid diversity (*Cervavitus, Procapreolus*) from the late Pannonian till the end of the Miocene [49].

### Biostratigraphy of the Sprendlingen Flora

The rich fossil seed and leaf flora discovered above the bone-bearing level was described by Meller [13]. The flora documents a riparian association of a mixed-mesophytic forest, with a dominance of *Salix*, *Ulmus* and Betulaceae. Stratigraphically most interesting elements are *Taxodium* cf. *hantkei*, *Fagus* cf. *heidingeri*, *Quercus kubinyi*, and *Daphnogene polymorpha*. This association shows strong similarities (as already noticed by Meller [13] to late Middle Miocene floras from the North Alpine Foreland Basin (NAFB) in Bavaria. In southern Germany these four plant taxa characterize localities such as Massenhausen ([50], [51]), Achldorf ([52], [51], [53]), and partly Lerch and Gumpersdorf 2 ([54], [55]), confined to the late Middle Miocene lithostratigraphic units *Obere Serie*, *Moldanubische Serie*, and *Südlicher Vollschotter*. In younger lithostratigraphic units like the *Hangendserie* (terminal Middle Miocene) or the early Late Miocene *Kobernaußerwald Schotter* and *Kohleton Serie* in Upper Austria, as well as in the Pannonian of the Vienna Basin the lauraceous taxon *Daphnogene* has already disappeared [55], [56], [57], [58], [59].

The exact chronostratigraphic position of the lithostratigraphic units *Obere Serie*, *Moldanubische Serie*, and *Südlicher Vollschotter* is still under investigation. However, their large mammal fauna with *Deinotherium levius* ( =  *D*. aff. *giganteum*) in Massenhausen [32], [50] and Achldorf [52], *Euprox furcatus* and advanced *Listridon splendens* in Massenhausen [50], [60], [61], as well as the lack of hipparionine horses in these localities, clearly indicate an age between 13 and 12 Ma.

### Palaeoclimatic Analysis of the Sprendlingen Flora

Based on comparisons with selected, climate sensitive Nearest Living Relatives (NLRs) of the palaeoflora from Sprendlingen Meller [13] estimated mean annual temperature (MAT) to be between 11–15°C and mean annual precipitation (MAP) to be between 1000 and 1200 mm. Additionally she estimated January temperatures to be around the freezing point, although she stated that the average temperatures may have been below 0°C for up to three months, and temperatures of the warmest month (WMMT) to be above 22°C. Later on Uhl et al. [62] estimated MAT to be at 13.6–15.8°C based on the Coexistence Approach [63], utilizing a large number of NLRs.

Here we re-analyzed the palaeoflora using the Coexistence Approach (CoA) with an updated database. Estimates for selected palaeoclimate parameters are presented in Table 1. These data generally support the overall climate interpretation of Meller [13] but for all temperature parameters slightly higher temperatures have been reconstructed with the CoA as compared to the more “intuitive” approach utilized by Meller [13]. MAP is only marginally higher with CoA, and CoA precipitation reconstructions for wettest (P_wet_), driest (P_dry_) and warmest (P_warm_) months suggest a moderate seasonality in precipitation during deposition of the clay lens.

**Table 1:** Selected climate parameters reconstructed with the CoA based on the Sprendlingen flora.

Climate parameter	CoA
MAT [°C]	14.1–14.5
WMMT [°C]	25.3–26.4
CMMT [°C]	0.6–1.7
MAP [mm]	1231–1237
P_wet_ [mm]	116–170
P_dry_ [mm]	43–43
P_warm_ [mm]	81–94

Mean annual temperature (MAT), temperature of the warmest month (WMMT), temperature of the coldest month (CMMT), mean annual precipitation (MAP), precipitation of the wettest month (P_wet_), precipitation of the driest month (P_dry_), precipitation of the warmest month (P_warm_).

The slightly warmer temperatures, as compared to Meller [13], fit well with the assumed late Middle Miocene age of the flora.

### Silicified Wood from Sprendlingen 2

**Figure 6:**
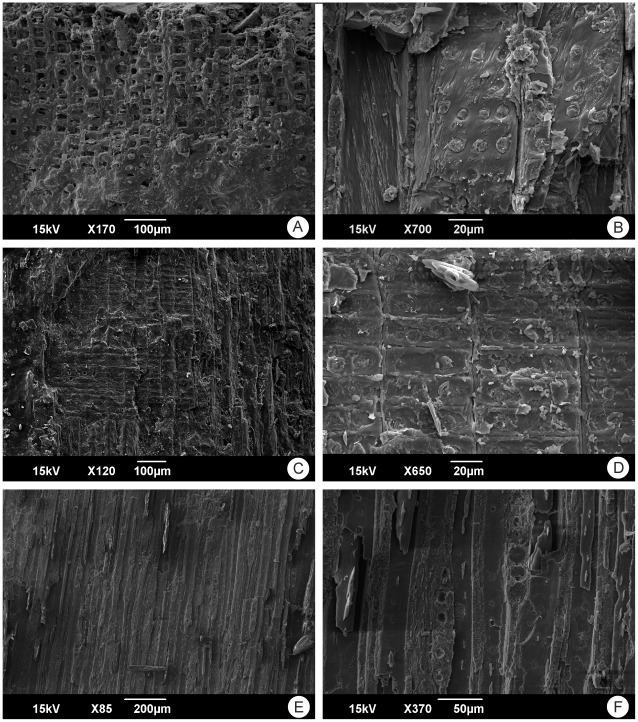
SEM images of the silicified Cupressaceae wood from the Eppelsheim Formation of Sprendlingen 2. A: tracheids (probably latewood) in cross section, in the upper part cell lumina are free of silica, whereas in the lower part of the images both cell walls and lumina have been silicified. B: tracheids with two to three (rarely four) seriate, oppositely arranged pits in radial view, a character typical for several members of the Cupressaceae [103]. C: tracheids with wood rays in radial view. D: close-up of crossfields with 2 pits per field. E: overview of wood with numerous wood rays in tangential view. F: close up of wood rays (each 6 cells high) in tangential view. Anatomical features observable in this specimen point to an affiliation to the (taxodioid) Cupressaceae (cf. [103]), although a more specific determination is not possible.

From the same level as the vertebrate remains a single piece of silicified wood has been recovered during excavations. Visible anatomic characters of the wood (Fig. 6) point to an affiliation with Cupressaceae. A more specific affiliation is not possible due to the lack of diagnostic characters. Cupressaceae are also known from the macroflora described by Meller [13], in particular taxa belonging to taxodioid Cupressaceae like *Taxodium* and probably *Sequoia*. Especially *Taxodium* is an element of the swamp vegetation, and was probably growing near the river banks. Silicified wood has also been reported from other localities, which are assumed to belong to the Eppelsheim Formation [64], but these woods have not been investigated in detail so far and it is thus not clear whether these specimens may represent much older (i.e. Late Paleozoic) material which has been reworked or not. Wagner [65] mentioned also silicified angiosperm wood from the Dinotheriensande, but nothing is known about the sedimentological context of these remains and unfortunately these remains have never been investigated in detail.

### Stratigraphic Distribution of Silicified Wood in the Neogene of Central Europe

Also the occurrence of silicified Cupressaceae wood within the bone-bearing level directly below the leaf flora in Sprendlingen 2 provides stratigraphic information. The silicification of wood is a permineralization process by silica solution or colloid [66], [67]. Silica saturated waters can be supplied under the influence of volcanic activities or intense silica weathering, which is most amplified under warm and humid climate. In southern Germany, the silicification of wood is a common process from the late Early Miocene up to the early Middle Miocene [68]. This corresponds to the warm and humid Miocene Climatic Optimum [69], [70]. The youngest silicified wood from the NAFB is about 14.5 Ma old, and associated with intensely weathered gravels [68]. Younger Middle Miocene sediments contain fresh feldspar grains (e.g. *Moldanubische Serie*, [71]) and fossil wood is always non-silicified (usually preserved as limonitic-filled holes, like in the middle part of the Sprendlingen 2 section). These data indicate that in Central Europe favorable climatic (weathering) conditions for the silicification of wood may have terminated by the end of the Miocene Climatic Optimum around 14 Ma, when mean annual temperatures in southern Germany decreased from between 18.6 and 20.8°C at around 15 Ma to below 16°C after 14 Ma [72]. Silicified wood in the bone-bearing level therefore indicates an age older than 14 Ma.

The only other period from which silicified wood is known in the source region of the Eppelsheim Formation is the Late Palaeozoic (i.e. the Late Carboniferous and the Early Permian; [73]) but wood from this period does definitively not belong to Cupressaceae as fossils belonging to this family are only known from Mesozoic and Cenozoic sediments (i.e from the Jurassic or even Middle Triassic onwards) [74].

In Eppelsheim, where the type locality of the Eppelsheim Formation is located, only limonitic woods have so far been discovered [75] but Wagner [65] mentioned the occurrence of silicified angiosperm wood from other localities belonging to the Eppelsheim Formation. Unfortunately no further details are known about this material.

## Discussion

### Biostratigraphy of the Fossil Horizons from Sprendlingen 2

The lower 150 cm of the Sprendlingen 2 section revealed rich fossil assemblages in two horizons: mammalian fossils and the silicified wood were found in the lowermost 45 cm of coarse sand to medium-grained gravel, leaf and seed flora originate from the upper 80 cm of clay.

Mammal fossils consist of about 300 isolated large mammal teeth and very few (<20) cranial and postcranial bones. A faunal list, including the number of specimens is given in Table 2. The majority of teeth belong to Rhinoceratidae (180 teeth), followed by Deinotheriidae (70 teeth), and Gomphotheriidae (49 teeth). Amphicyonidae are represented by two teeth and Chalicotheriidae only by a middle phalange from the third digit of the hand, which is identical in morphology and dimensions to *Anisodon grande* described by Zapfe [76] from Devinska Nova Ves Fissure and Korotkevich and Suliminski [77] from Przeworno 2. Cervids are represented by the described antlers only.

**Table 2:** Faunal list of Sprendlingen 1 (after Wagner 1947) and Sprendlingen 2 (this study).

Sprendlingen 1	Sprendlingen 2
*Deinotherium giganteum* (3)	Amphicyonidae indet. (2)
*Zygolophodon turicensis* (1)	*Deinotherium bavaricum* (51)
*Tetralophodon longirostris* (4)	*Deinotherium levius* (15)
*Tapirus priscus* (3)	*Gomphotherium angustidens* (35)
*Aceratherium incisivum* (8)	*Zygolophodon turicensis* (10)
*Dihoplus scheiermacheri* (1)	*Tetralophodon longirostris* (4)
*Chalicotherium goldfussi* (1)	*Brachypotherium* sp. (120)
*Hippotherium primigenium* (5)	*Aceratherium* s.l. sp. (60)
*Propalaeochoerus palaeochoerus* (3)	*Anisodon grande* (1)
*Euprox* vel *Amphiprox* (3)	*Dicrocerus elegans* (2)
	*Paradicrocerus elegantulus* (1)

The approximate number of individuals (Sprendlingen 1) and specimens (Sprendlingen 2) is given in brackets.

This mammal collection found at the base of the Eppelsheim Formation is exceptional, because no other locality in Europe outside the *Dinotheriensande* shows a comparable faunal association. Elsewhere, the individual species recorded in Sprendlingen 2 in one layer, occur in different stratigraphic periods only. This indicates the existence of at least two biostratigraphic levels within this basal bone-bearing layer. The older adheres both deer species, *Dicrocerus elegans* and *Paradicrocerus elegantulus,* the deinothere *Deinotherium bavaricum,* the gomphothere *Gomphotherium angustidens,* as well as the silicified *Taxodioxylon* wood. This association is restricted to the early Middle Miocene (Langhian, Early Badenian). In southern Germany and Central Europe in general (but also in France, [78]) these four mammal species co-occur between 15.3 and 14.2 Ma, together with abundant silicified wood. The biostratigraphic position of the gomphothere *Zygolophodon turicensis* and the chalicothere *Anisodon grande* is somewhat ambiguous because they occur throughout the Middle Miocene. In contrast, both, the deinothere *Deinotherium levius* and the gomphothere *Tetralophodon longirostris* coexist during the late Middle Miocene. This stratigraphic position is supported by the late Middle Miocene age of the flora from the overlying clays. There is no biostratigraphic indication for a Late Miocene age in the basal layer of the Eppelsheim Formation at Sprendlingen 2.

These results suggest, that at least the lower part of the Sprendlingen 2 section was buried during the late Middle Miocene, thereby reworking older (early Middle Miocene) fluvial sediments containing the typical Langhian/Early Badenian fauna and flora.

### Biostratigraphy of Older Collections from Sprendlingen (Sprendlingen 1)

Interestingly, beside the here described large fossil sample collected by V. Knörzer and A. and H. Stapf in one fluvial channel deposit during the early 1980ies (Sprendlingen 2), Wagner [65]: 172 mentioned another fossil collection form Sprendlingen, probably destroyed during World War II (Sprendlingen 1). He describes an accumulation deposited likewise in a single fluvial channel at the base of the *Dinotheriensande*. Over 400 teeth and bones excavated on 25 square meters reveal according to Wagner [65] the following species (number of individuals in brackets): *Deinotherium giganteum* (3), *Tetralophodon longirostris* (4), *Zygolophodon turicensis* (1), *Tapirus priscus* (3), *Aceratherium incisivum* (8), *Dihoplus scheiermacheri* (1), *Chalicotherium goldfussi* (1), *Hippotherium primigenium* (5), *Propalaeochoerus palaeochoerus* (3), and *Euprox* vel *Amphiprox* (3). Besides the two gomphotheres this sample differs fundamentally from the assemblage of Sprendlingen 2 described above, sampled more than 40 years later and probably not more than a few hundred meters away. Especially with *T. priscus, A. incisivum, D. schleiermacheri, H. primigenium* and *P. palaeochoerus* the Sprendlingen 1 association can clearly be correlated to the early Late Miocene (early Vallesian, MN9).

Thus, faunal assemblages Sprendlingen 1 and 2 do not only indicate stratigraphic inhomogenity for the Eppelsheim Formation, but even show different ages for its erosive base over rather short distances.

### Miocene Deer Diversity in Central Europe

**Figure 7:**
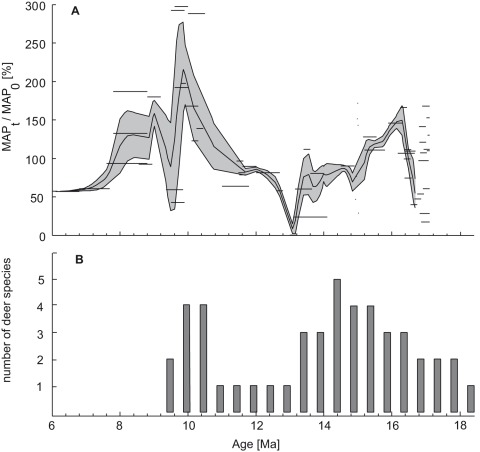
Miocene humidity and deer diversity in Central Europe. A: Relative mean annual precipitation (MAP_t_/MAP_0_ ×100%) for Central and Eastern Europe during the Miocene (from [70]; horizontal black bars represent the raw data with age uncertainties). B: Species diversity (gamma diversity) of Miocene deer in Central Europe.

The stratigraphic pattern (Fig. 5) of Miocene deer indicate, that two periods of high diversity existed in Central Europe with up to four or five contemporaneous deer species (gamma-diversity). The first period is during the Langhian or Early Badenian (∼15.3 to ∼14.2 Ma) with four to five species, the second period occurs during the early Tortonian or early and middle Pannonian (∼10.5 to 10 Ma) with up to four species. From 13.5 Ma till ∼10.5 Ma we observe a crisis (drop) in cervid diversity, characterized by *Euprox furcatus* as the only deer species. The resulting double-peaked cervid diversity pattern during the Miocene shows interesting similarities to the regional humidity evolution in Central Europe (Fig. 7; [70]). Cervid diversity peaks correlate with high precipitation (150% or more relative to recent) and an intensified hydrologic cycle (washhouse climate periods sensu Böhme et al. [79]), whereas diversity drops occur when rainfall values decrease (on longer timescales) below present-day values. This correspondence suggests an intimate relation between the structures of forest environments and the evolution of Cervidae.

### Implications for European Large Mammal Chronology

As mentioned above the discovery of a mixed Middle Miocene mammal association in the Eppelsheim Formation of Sprendlingen 2 demonstrates the stratigraphic inhomogeneity of vertebrate fossils from the *Dinotheriensande* in Rheinhessen. This was already suggested by some earlier authors (so-called *Mischfauna* of Haupt [80] and Klähn [81]), but subsequently disputed by others [82], [83], [84]. Haupt [17] studied the Cervidae and stated that *Amphiprox anocerus* is the ‘real’ Late Miocene *Dinotheriensand* deer, whereas *Euprox furcatus, Heteroprox larteti* and *Dicrocerus elegans* have been reworked from older deposits.

Moreover, looking in detail on the faunal list from the type locality Eppelsheim, and other localities of this formation [17], [65], [32], [85], [86], [83], [87], [88], [89], [10], [90], [6], several species can be noticed, which typically occur in the Middle Miocene. These are, besides the proboscidians *Deinotherium bavaricum, D. levius, Zygolophodon turicensis* and *Gomphotherium angustidens,* the pigs *Hyotherium soemmeringi, Conohyus simorrensis, Parachleuastochoerus huenermanni,* and *Listriodon splendens,* the palaeomerycid *Palaeomeryx eminens,* the deers *Heteroprox larteti* and *Euprox furcatus,* as well as the crocodile *Diplocynodon*. Except *D. levius*, *P. huenermanni*, *L. splendens,* and *E. furcatus* all these taxa co-occur with *Dicrocerus elegans* and *Paradicrocerus elegantulus* in the NAFB during the early Middle Miocene. *Listriodon splendens* and *E. furcatus* have their First Occurrence Date in Central Europe at ∼14.2 Ma in Klein-Hadersdorf ([20], [60]), *P. huenermanni* between 14 and 13 Ma in Breitenbrunn ([89], [41], and own data), and *D. levius* between 13 and 12.5 Ma in La Grive, and respectively in early Sarmatian sediments of Austria [91], [34]. These species (except the rare *P. huenermanni*) represent a very characteristic association of the late Middle Miocene in Central Europe [92], [91].

Based on these data we therefore can differentiate at least three biochronologic levels within the large mammals of the classic *Dinotheriensand* fauna (excluding Dorn-Dürkheim) (Table 3), corresponding to the early Middle Miocene (MN5 and MN6; late Orleanian to early Astaracian), late Middle Miocene (MN7/8; late Astaracian), and early Late Miocene (MN9; early Vallesian), covering at least six million years. Small mammals may even point to late Vallesian (MN10) or early Turolian (MN11) ages [10], [93], [94].

**Table 3:** Large herbivore mammals and crocodiles from the Eppelsheim Formation and their biostratigraphy.

early Middle Miocene (MN5, 6)	late Middle Miocene (MN7/8)	early Late Miocene (MN9)
*Deinotherium bavaricum*	*Deinotherium levius*	*Deinotherium giganteum*
*Gomphotherium angustidens*	*Tetralophodon longirostris*	*Tetralophodon longirostris*
*Zygolophodon turicensis*	*Zygolophodon turicensis*	*Stegotetralophodon gigantorostris*
*Anisodon grande*	*Anisodon grande*	*Chalicotherium goldfussi*
*Anchitherium aurelianense*	*Anchitherium* sp. indet.	*Tapirus priscus*
*Hyotherium soemmeringi*	*Conohyus simorrensis*	*Tapirus antiquus*
*Dicrocerus elegans*	*Parachleuastochoerus huenermanni*	*Hippotherium primigenium*
*Paradicrocerus elegantulus*	*Listriodon splendens*	*Propalaeochoerus palaeochoerus*
*Palaeomeryx eminens*	*Palaeomeryx eminens*	*Microstonyx antiquus*
*Diplocynodon* sp.	*Dorcatherium naui*	*Dorcatherium naui*
	*Euprox furcatus*	*Euprox dicranocerus*
		*Amphiprox anocerus*
		*Miotragocerus pannoniae*

Note that *Zygolophodon turicensis, Anisodon grande,* and *Palaeomeryx eminens* occurred throughout the Middle Miocene, *Tetralophodon longirostris* and *Dorcatherium naui* in the late Middle and early Late Miocene.

Our understanding of the Central European early Vallesian as a ‘phase of supersaturation’ [95]: 441 in biodiversity [96] is biased by large-mammal faunas from the Eppelsheim Formation (proto-Rhine), but also from the Hollabrunn-Mistelbach Formation (proto-Danube) in Lower Austria where complex faunal mixing was documented only recently [46]. Therefore, the result of stratigraphically mixed and inhomogeneous *Dinotheriensande* has sever implications not only for the understanding of basin evolution (see below), biostratigraphy and palaeobiology (e.g. the problem of Middle Miocene superstites in the Late Miocene and thereby the obliteration of turnover events), but also for macro-scaled palaeoenvironmental studies, like the concept of a permanently stable, humid and forested but ‘strangely elusive’ [95]: 444 Central European bioprovince. Although some Central European early Vallesian sites are certainly stratigraphically unbiased (e.g. Höwenegg, Rudabanya), we argue that only a careful re-examination of old collections, together with fieldwork evidence and new well documented excavations (like those accomplished by the Forschungsinstitut Senckenberg and the Natural History Museum Mainz since 1996 in Eppelsheim [3], [97], [11]) can help clarifying the various palaeobiologic events around the Middle to Late Miocene transition.

### Implications for Lithostratigraphy and Palaeohydrology of the Mainz Basin

Our biostratigraphic results imply that the Eppelsheim Formation correlates chronostratigraphically to the early Middle to early Late Miocene. This has consequences for the regional stratigraphic correlation, and the understanding of basin development and palaeohydrologic evolution of the Mainz Basin and the Rhine Graben in general.

**Figure 8:**
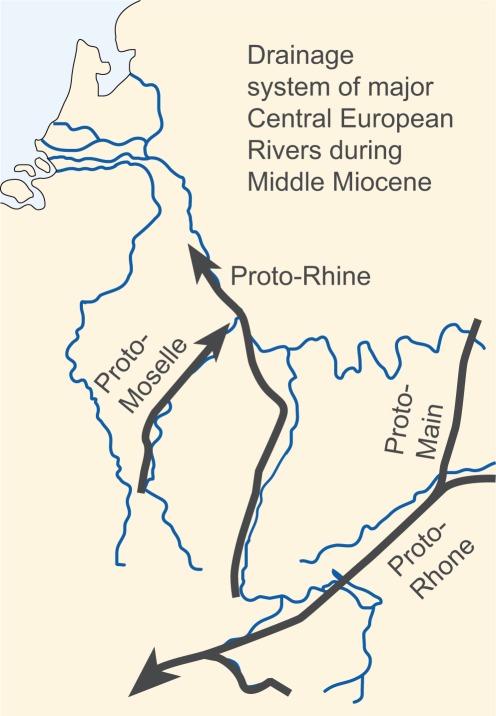
The course of main Central European Rivers during the early Middle Miocene (Langhian). Modified after [104].

Adjacent to the Mainz Basin, in the Upper Rhine Graben and the Hanau Basin, a significant change in sedimentation style occurred between the late Early Miocene Praunheim and Staden Formations (Fig. 1), with a switch from brackish-lacustrine, carbonate dominated sedimentation, to siliciclastic fluvial sedimentation with paleosols [98]. Along this transition, dated to about 17 Ma, the carbonate sedimentation (*Kalktertiär*) ended in this area. The siliciclastic Staden Formation and the lithologically practically identical Bockenheim Formation are separated by the tholeiitic Untermain-Basalt Formation, which gave radiometric K-Ar ages between 16.3 and 14.8 Ma [99]. Our stratigraphic results from the Eppelsheim Formation of the Mainz Basin point to a more-or-less contemporary onset of siliciclastic, fluvial sedimentation in the Mainz Basin, and the Upper Rhine Graben and Hanau Basin around the Early to Middle Miocene transition. The early Middle Miocene (16 to 15 Ma) onset of fluvial sedimentation in the Mainz Basin shifts back the origination of the proto-Rhine (e.g. the Kaiserstuhl-Rhine of [7], the first drainage system connecting Upper and Middle Rhine Graben with the Lower Rhine Embayment) by some 5 million years (Fig. 8). This age fits with the oldest heavy-mineral deposit reflecting an Upper Rhine Graben source area in the Lower Rhine Embayment [100]. In the Mainz Basin, low basin subsidence during the Neogene [97] prevents an accumulation of thick fluvial packages and rather facilitates a cannibalistic reworking during younger stages of Rhine River sedimentation.

## Materials and Methods

The Sprendlingen 2 fossil material studied here is stored in the Naturhistorisches Museum Mainz (MNHM) and the Paläontologisches Museum Nierstein (SSN). It has been excavated in the early 1980ies by Volker Knörzer, Arnulf Stapf and Harald Stapf.

### Institutional abbreviations

NHMM: Naturhistorisches Museum Mainz

NMA: Naturmuseum Augsburg

SSN: Paläontologisches Museum Nierstein

ML: Museum of Lyon

BSPG: Bayerische Staatssammlung für Paläontologie und Geologie

### Palaeoclimate Analysis

The Sprendlingen macroflora was analysed using the Coexistence Approach, a technique based on comparisons with Nearest Living Relatives [63]. For application of the Coexistence Approach we followed standard protocols using an updated version of the PALAEOFLORA database [101], which led to slightly different MAT estimates for Sprendlingen as published by Uhl et al. [62] based on an older version of this database.

### Anatomical Analysis of Silicified Wood

Pieces of the wood were investigated closely under the binocular to identify areas which are likely to provide anatomical information under the SEM. Small fragments of the wood which were identified as suitable samples were extracted mechanically and mounted on standard stubs with LeitC (Plano GmbH, Germany), and subsequently examined with the aid of a JEOL JSM 6490 LV SEM (at 15 kV) at the Senckenberg Forschungsinstitut und Naturmuseum Frankfurt.

## Supporting Information

Table S1 Metric data of upper and lower P3-M1 molars of *Deinotherium* species as used in Fig. 4 of the manuscript.
(DOCX)

Table S2 List of cervid localities from Central Europe and the reference regarding taxonomy and/or biostratigraphy.
(DOCX)

## References

[1] Cuvier G (1812). Recherches sur les ossemens fossiles de quadrupèdes ou l'on rétablit les caractères de plusieurs espèces d’animaux que les révolutions du globe paraoissent avoir détruites..

[2] Goethe JWv (1816). Kunst und Alterthum in den Rhein- und Maingegenden..

[3] Franzen JL (2000). Auf dem Grunde des Urrheins –Ausgrabungen bei Eppelsheim.. Natur und Museum.

[4] Sandberger F (1853). Untersuchungen über das Mainzer Tertiärbecken und dessen Stellung im geologischen Systeme..

[5] Grimm MC (2005). Beiträge zur Lithostratigraphie des Paläogens und Neogens im Oberrheingebiet (Oberrheingraben, Mainzer Becken, Hanauer Becken).. Geologisches Jahrbuch Hessen.

[6] Franzen JL, Grimm KI (2011). 5.2.7.3 Eppelsheim Formation.. Stratigraphie von Deutschland IX Tertiär, Teil 1: Oberrheingraben und benachbarte Tertiärgebiete.

[7] Schirmer W, Schirmer W (2003). Stadien der Rheingeschichte.. Landschaftsgeschichte im europäischen Rheinland.

[8] Schäfer P (2012). Mainzer Becken Stratigraphie - Paläontologie - Exkursionen; Rothe P, editor..

[9] Franzen JL, Storch G, Agustì J, Rook L, Andrews P (1999). Late Miocene mammals from Central Europe.. The evolution of Neogene terrestrial ecosystems in Europe.

[10] Franzen JL, Fejfar O, Storch G (2003). First micromammals (Mammalia, Soricomorpha) from the Vallesian (Miocene) of Eppelsheim, Rheinhessen (Germany).. Senckenbergiana lethaea.

[11] Sommer J, Kullmer O, Holzförster F, Lutz H (2009). Die obermiozänen Dinotheriensande (Eppelsheim-Formation) bei Eppelsheim/Rheinhessen unter dem Gesichtspunkt neuer sedimentologischer, taphonomischer und paläoökologischer Ergebnisse.. Mainzer naturwissenschaftliches Archiv.

[12] Wagner W (1973). Die unterpliozänen Dinotherien-Sande und ihre Fauna im Gebiet des Blattes 6114 Wörrstadt (Mainzer Becken).. Mainzer Geowissenschaftliche Abhandlungen.

[13] Meller B (1989). Eine Blatt-Flora aus den Obermiozänen Dinotheriensanden (Vallesium) von Sprendlingen (Rheinhessen).. Documenta naturae.

[14] Radtke G, Martini E (2008). Neudefinition von stratigraphischen Einheiten im Tertiär des Mainzer und Hanauer Beckens (W-Deutschland, Miozän): Frankfurt-Formation [ =  Obere Hydrobien-Schichten].. Geologisches Jahrbuch Hessen.

[15] Azanza B (1993). Sur la nature des appendices frontaux des cervidés (Artiodactyla, Mammalia) du Miocène inférieur et moyen. Remarques sur leur systématique et leur phylogénie. On the nature of the frontal appendages in Lower-Middle Miocene deer (Artiodactyla, Mammalia). Contribution to their systematics and phylogeny.. Comptes rendus de l’Académie des sciences, Série 2.

[16] Ginsburg Lo, Azanza B (1991). Présence de bois chez les femelles du cervidé miocène *Dicrocerus elegans* et remarques sur le problème de l’origine du dimorphisme sexuel sur les appendices frontaux des cervidés. Antlers in females of the Miocene deer *Dicrocerus elegans* and some remarks on the origin of the sexual dimorphism in the deer cranial appendices.. Comptes rendus de l’Académie des sciences, Série 2.

[17] Haupt O (1935). Bemerkungen über die Hirsche aus dem Dinotheriensand Rheinhessens.. Notizblatt der Hessischen Geologischen Landesanstalt.

[18] Rössner GE (2010). Systematics and palaeoecology of the Ruminantia (Artiodactyla, Mammalia) community from Sandelzhausen (Early/Middle Miocene) in the German Molasse Basin.. Paläontologische Zeitschrift.

[19] Stehlin HG (1939). *Dicrocerus elegans* LARTET und sein Geweihwechsel.. Eclogae geologicae Helvetiae.

[20] Thenius E (1948). Zur Kenntnis der fossilen Hirsche des Wiener Beckens, unter besonderer Berücksichtigung ihrer stratigraphischen Bedeutung.. Annalen des Naturhistorischen Museums in Wien.

[21] Thenius E (1950). Die tertiären Lagomeryciden und Cerviden der Steiermark.. Sitzungsbericht der Österreichischen Akademie der Wissenschaften, Mathematisch-naturwissenschaftlichen Klasse I.

[22] Stehlin HG (1937). Bemerkungen über die miocaenen Hirschgenera *Stephanocemas* und *Lagomeryx*.. Verhandlungen der Naturforschenden Gesellschaft in Basel.

[23] Ginsburg Lo, Cheneval J, Janvier P, Pouit D, Sen S (2000). Les Vertébrés des sables continentaux d’âge orléanien inférieur (MN 3) de Mauvières à Marcilly-sur-Maulne (Indre-et-Loire), La Brosse à Meigné-le-Vicomte (Maine-et-Loire) et Chitenay (Loir-et-Cher).. Geodiversitas.

[24] Rössner GE (1995). Odontologische und schädelanatomische Untersuchungen.. Münchner Geowissenschaftliche Abhandlungen A.

[25] Stehlin HG (1928). Bemerkungen über die Hirsche von Steinheim am Albuch.. Eclogae geologicae Helvetiae.

[26] Ginsburg Lo, Crouzel F (1976). Contribution à la connaissance d’*Heteroprox larteti* (Filhol) : Cervidé du Miocène européen.. Bulletin du Muséum national d’histoire naturelle Sciences de la terre.

[27] Gentry AW (1994). The Miocene differentiation of Old World Pecora (Mammalia).. Historical Biology.

[28] Roger O (1904). Wirbelthierreste aus dem Dinotheriensande der bayerisch-schwäbischen Hochebene (V. Theil).. Bericht des Naturwissenschaftlichen Vereins für Schwaben und Neuburg in Augsburg.

[29] Rössner GE (2006). A community of Middle Miocene Ruminantia (Mammalia, Artiodactyla) from the German Molasse Basin.. Palaeontographica A.

[30] Azanza B, Ginsburg Lo (1997). A revision of the large lagomerycid artiodactyls of Europe.. Palaeontology.

[31] Weinsheimer O (1883). Über Dinotherium giganteum Kaup.. Paläontologische Abhandlungen.

[32] Gräf IE (1957). Die Prinzipien der Artbestimmung bei *Dinotherium*.. Palaeontographica A.

[33] Bergounioux F-M, Crouzel F (1962). Les Déinothéridés d’Europe.. Annales de Paléontologie.

[34] Ginsburg L, Chevrier F (2001). Les Dinothères du bassin de la Loire et lévolution du genre *Deinotherium* en France.. Symbioses.

[35] Huttunen K (2002). Deinotheriidae (Proboscidea, Mammalia) dental remains from the Miocene of Lower Austria and Burgenland.. Annalen des Naturhistorischen Museum Wien A.

[36] Tobien H (1988). Contributions a l’étude du gisement miocène supérieur de Montredon (Herault)..

[37] Kaup JJ (1829). Deinotherium giganteum, eine Gattung der Vorwelt aus der Ordnung der Pachydermen, aufgestellt und beschrieben.. Isis.

[38] Mottl M (1958). Proboscidierfunde aus dem Sarmat der Steiermark.. Mitteilungen des Museums für Bergbau, Geologie und Technik des Landesmuseum Joanneum.

[39] Mottl M (1969). Bedeutende Proboscidier-Neufunde aus dem Altpliozän (Pannonien) Südost-Österreichs.. Österreichische Akademie der Wissenschaften, mathematisch-naturwissenschaftliche Klasse, Denkschriften.

[40] Harzhauser M, Piller W (2004). Integrated stratigraphy of the Sarmatian (Upper Middle Miocene) in the western Central Paratethys.. Stratigraphy.

[41] Seehuber U (2008). Litho- und biostratigraphische Untersuchungen in der Oberen Süßwassermolasse in der Umgebung von Kirchheim in Schwaben [Doctoral Thesis]..

[42] Abdul Aziz H, Böhme M, Rocholl A, Zwing A, Prieto J (2008). Integrated stratigraphy and 40Ar/39Ar chronology of the Early to Middle Miocene Upper Freshwater Molasse in eastern Bavaria (Germany).. International Journal of Earth Sciences.

[43] Abdul Aziz H, Böhme M, Rocholl A, Prieto J, Wijbrans J (2010). Integrated stratigraphy and 40Ar/39Ar chronology of the early to middle Miocene Upper Freshwater Molasse in western Bavaria (Germany).. International Journal of Earth Sciences.

[44] Prieto J, Böhme M, Maurer H, Heissig K, Abdul Aziz H (2009). Biostratigraphy and sedimentology of the Fluviatile Untere Serie (Early and Middle Miocene) in the central part of the North Alpine Foreland Basin: implications for palaeoenvironment and climate.. International Journal of Earth Sciences.

[45] Kälin D, Kempf O (2009). High-resolution stratigraphy from the continental record of the Middle Miocene Northern Alpine Foreland Basin of Switzerland.. Neues Jahrbuch für Geologie und Paläontologie, Abhandlungen.

[46] Harzhauser M, Daxner-Höck G, Göhlich UB, Nagel D (2011). Complex faunal mixing in the early Pannonian palaeo-Danube Delta (Late Miocene, Gaweinstal, Lower Austria).. Annalen des Naturhistorischen Museum Wien A.

[47] Gross M, Böhme M, Prieto J (2011). Gratkorn: A benchmark locality for the continental Sarmatian s.str. of the Central Paratethys.. International Journal of Earth Sciences.

[48] Paulissen WE, Luthi SM, Grunert P, Coric S, Harzhauser M (2011). Integrated high-resolution stratigraphy of a Middle to Late Miocene sedimentary sequence in the central part of the Vienna Basin.. Geologica Carpathica.

[49] Korotkevich O (1988). Genesis of the Hipparion-Fauna of Eastern Europe..

[50] Jung W (1963). Blatt- und Fruchtreste aus der Oberen Süßwassermolasse von Massenhausen, Kreis Freising (Oberbayern).. Palaeontographica B.

[51] Knobloch E (1986). Die Flora aus der Oberen Süßwassermolasse von Achldorf bei Vilsbiburg (Niederbayern).. Documenta naturae.

[52] Jung W (1970). Eine reiche Fundstelle obermiozäner Pflanzenreste in der Oberen Süßwassermolasse Südbayerns.. Neues Jahrbuch für Geologie und Paläontologie, Monatshefte.

[53] Knobloch E (1988). Neue Ergebnisse zur Flora aus der Oberen Süßwassermolasse von Aubenham bei Ampfing (Krs. Mühldorf am Inn).. Documenta naturae.

[54] Jung W (1968). Pflanzenreste aus dem Jungtertiär Nieder- und Oberbayerns und deren lokalstratigraphische Bedeutung.. Berichte des naturwissenschaftlichen Vereins Landshut.

[55] Jung W, Mayr H (1980). Neuere Funde zur Biostratigrphie der Oberen Süßwassermolasse Süddeutschlands und ihre palökologische Deutung.. Mitteilungen der Bayerischen Staatssammlung für Paläontologie und historische Geologie.

[56] Jung W (1984). Die Florenentwickung in der Bayerischen Molasse.. Berichte des naturwissenschaftlichen Vereins Landshut.

[57] Jung W (1986). Ein Beitrag zur paläobotanischen Charakterisierung der „Jüngeren Serie“ der Oberen Süßwasser-Molasse Südbayerns.. Mitteilungen der Bayerischen Staatssammlung für Paläontologie und historische Geologie.

[58] Kovar-Eder J (1988). Obermiozäne (Pannone) Floren aus der Molassezone Österreichs.. Beiträge zur Paläontologie Österreichs.

[59] Meller B, Egger H, Rupp C (2007). Die Fazies der Braunkohle führenden obermiozänen Sedimente des Hausrucks (Molassebecken, Oberösterreich) aufgrund paläobotanisch-paläoökologischer Untersuchungen.. Beiträge zur Geologie Oberösterreichs: ATA Geologische Bundesanstalt 2007.

[60] van der Made J (1996). Listriodontinae (Suidae, Mammalia), their evolution, systematics and distribution in time and space.. Contributions to Tertiary and Quaternary Geology.

[61] Eronen JT, Rössner GE (2007). Wetland Paradise Lost: Miocene Community Dynamics in Large Herbivore Mammals from the German Molasse Basin.. Ecology and Evolutionary Research.

[62] Uhl D, Klotz S, Traiser C, Thiel C, Utescher T (2007). Paleotemperatures from fossil leaves – a European perspective.. Palaeogeography, Palaeoclimatology, Palaeoecology.

[63] Mosbrugger V, Utescher T (1997). The coexistence approach – a method for quantitative reconstructions of Tertiary terrestrial palaeoclimate data using plant fossils.. Palaeogeography, Palaeoclimatology, Palaeoecology.

[64] Grimm KI, Grimm MC, Grimm KI, Grimm MC, Neuffer FO, Lutz H (2003). Geologischer Führer durch das Mainzer Tertiärbecken.. Die fossilen Wirbellosen des Mainzer Tertiärbeckens.

[65] Wagner W (1947). Das Gebiet des unterpliozänen Ur-Rheins in Rheinhessen und seine Tierwelt.. Die Naturwissenschaften.

[66] Scurfield G, Segnit ER (1984). Petrification of Wood by Silica Minerals.. Sedimentay Geology.

[67] Mustoe GE (2003). Microscopy of Silicified Wood.. Microscopy Today.

[68] Böhme M, Bruch AA, Selmeier A (2007). The reconstruction of Early and Middle Miocene climate and vegetation in Southern Germany as determined from the fossil wood flora.. Palaeogeography, Palaeoclimatology, Palaeoecology.

[69] Böhme M (2003). The Miocene Climatic Optimum: evidence from ectothermic vertebrates of Central Europe.. Palaeogeography, Palaeoclimatology, Palaeoecology.

[70] Böhme M, Winklhofer M, Ilg A (2011). Miocene precipitation in Europe: Temporal trends and spatial gradients.. Palaeogeography, Palaeoclimatology, Palaeoecology.

[71] Stiefel J (1957). Beitrag zur Gliederung der Oberen Süßwassermolasse in Niederbayern.. Geologisches Jahrbuch, Beihefte.

[72] Ivanov M, Böhme M (2011). Snakes from Griesbeckerzell (Langhian, Early Badenian), North Alpine Foreland Basin (Germany), with comments on the evolution of snake faunas in Central Europe during the Miocene Climatic Optimum.. Geodiversitas.

[73] Kerp H, Noll R, Uhl D, Schindler T, Heidtke UHJ (2007). Vegetationsbilder aus dem saarpfälzischen Permokarbon.. Kohlesümpfe, Seen und Halbwüsten - Dokumente einer rund 300 Millionen Jahre alten Lebewelt zwischen Saarbrücken und Mainz.

[74] Taylor TN, Taylor EL, Krings M (2009). Paleobotany: The Biology and Evolution of Fossil Plants..

[75] Franzen JL, Fejfar O, Storch G, Wilde V (2003). Eppelsheim 2000 – New discoveries at a classic locality.. Deinsea - Annual of the Natural History Museum Rotterdam.

[76] Zapfe H (1979). *Chalicotherium grande* (Blainv.) aus der miozänen Spaltenfüllung von Neudorf an der March (Dvinská Nová Ves), Tschechoslowakei.. Neue Denkschriften des Naturhistorischen Museums Wien.

[77] Korotkevich EL, Suliminski A (1990). A new chalicotherian from the Miocene karst in Poland (Mammalia, Badenian).. Acta Palaeontologica Polonica.

[78] Ginsburg L (2000). Chronologie des dépôts miocènes du Blésois à la Bretagne.. Symbioses.

[79] Böhme M, Ilg A, Winklhofer M (2008). Late Miocene “washhouse” climate in Europe.. Earth and Planetary Science Letters.

[80] Haupt O (1914). Die Mischfauna der rheinhessischen Dinotheriensande und ihre Bedeutung für das Alter derselben..

[81] Klähn H (1931). Rheinhessisches Pliozän besonders Unterpliozän im Rahmen des Mitteleuropäischen Pliozäns.. Geologische und Paläontologische Abhandlungen, N F.

[82] von Koenigswald R (1929). Bemerkungen zur Säugetierfauna des rheinhessischen Dinotheriensandes.. Senckenbergiana.

[83] Tobien H (1980). A Note on the Mastodont Taxa (Proboscidea, Mammalia) of the ^?^Dinotheriensande” (Upper Miocene, Rheinhessen, Federal Republic of Germany).. Mainzer Geowissenschaftliche Abhandlungen.

[84] Tobien H (1983). Bemerkungen zur Taphonomie der spättertiären Säugerfauna aus den Dinotheriensanden Rheinhessens..

[85] Tobien H (1953). Miotragocerus STROMER (Bovidae, Mamm.) aus den unterpliozänen Dinotheriensande Rheinhessens.. Notizblatt Hessisches Landesamt für Bodenforschung.

[86] Tobien H (1961). Palaeomeryx eminens H. v. M. (Cervoidea, Mamm.) aus dem unterpliozänen Dinotheriensanden Rheinhessens.. Neues Jahrbuch für Geologie und Paläontologie, Monatshefte.

[87] Hünermann KA (1968). Die Suidae (Mammalia, Artiodactyla) aus den Dinotheriensanden (Unterpliozön: Pont) Rheinhessens (Südwestdeutschland).. Schweizer Paläontologische Abhandlungen.

[88] Abusch-Siewert (1983). Gebissmorphologische Untersuchungen an Eurasiatischen Anchitherien (Equidae, Mammalia) unter besonderer Berücksichtigung der Fundstelle Sandelzhausen.. Courier Forschunginstitut Senckenberg.

[89] Heissig K (1989). *Conohyus huenermanni* n.sp., eine kleine Schweineart aus der Oberen Süßwassermolasse Bayerns.. Mitteilungen der Bayerischen Staatssammlung für Paläontologie und historische Geologie.

[90] Sommer J (2007). Sedimentologie, Taphonomie und Paläoökologie der miozänen Dinotheriensande von Eppelsheim/Rheinhessen..

[91] Mottl M (1970). Die jungtertiären Säugetierfaunen der Steiermark, Südost-Österreichs.. Mitteilung des Museums für Bergbau, Geologie und Technik am Landesmuseum ?Joanneum” Graz.

[92] Thenius E (1960). Die jungtertiären Wirbeltierfaunen und Landfloren des Wiener Beckens und ihre Bedeutung für die Neogenstratigraphie.. Mitteilungen der Osterreichische Geologische Gesellschaft.

[93] Ziegler R (2006). Insectivores (Lipotyphla) and bats (Chiroptera) from the Late Miocene of Austria.. Annalen des Naturhistorischen Museum Wien A.

[94] Kullmer O, Morlo M, Sommer J, Lutz H, Engel T (2008). The Second Specimen of Simocyon Diaphorus (Kaup, 1832) (Mammalia, Carnivora, Ailuridae) from the Type–Locality Eppelsheim (Early Late Miocene, Germany).. Journal of Vertebrate Paleontology.

[95] Fortelius M, Werdelin L, Andrews P, Bernor RL, Gentry A, Bernor RL, Fahlbusch V, Mittmann HW (1996). Provinciality, diversity, turnover, and paleoecology in land mammal faunas of the later Miocene of western Eurasia.. The evolution of Western Eurasian Neogene mammal faunas.

[96] Casanovas-Vilar I, Moyà-Solà S, Agustì J, Köhler M, Elewa A (2005). The geography of a faunal turnover: tracking the Vallesian Crisis.. Migration in organisms: climatology, geography, ecoology.

[97] Holzförster F, Sommer J, Kullmer O, Lutz H (2008). Der Obermiozäne Ur-Rhein bei Eppelsheim (Rheinhessen) und sein Bezug zurTektonik des Mainzer Beckens.. Mainzer naturwissenschaftliches Archiv.

[98] Radtke G, Kümmerle E (2004). Neudefinition von fünf stratigraphischen Einheiten im Tertiär (Miozän) des Hanauer Beckens und des Oberrheingrabens (Deutschland): Niederrad- bis Bockenheim-Formation.. Geologisches Jahrbuch Hessen.

[99] Fuhrmann U, Lippolt HJ (1987). K-Ar-Datierungen an Maintrapp-Basalten aus Bohrungen in Frankfurt a. M. nach der 40Ar/39Ar-Stufenentgasungstechnik.. Geologisches Jahrbuch Hessen.

[100] Boenigk W (1982). Einfluss des Rheingrabensystems auf die Flussgeschichte des Rheins.. Rheinische Zeitschrift für Geomorphologie.

[101] Utescher T, Mosbrugger V (1997). PALAEOFLORA – Data base for palaeoclimate reconstructions using the Coexistence Approach.

[102] Franzen JL (2006). Ein Paradies für Säuger? Das Obermiozän Mitteleuropas.. Biologie in unserer Zeit.

[103] Richter HG, Grosser D, Heinz I, Gasson PE (2004). IAWA list of microscopic features for softwood identification..

[104] Preusser F (2008). Characterization and evolution of the River Rhine system.. Netherlands Journal of Geoscience.

