# Supplementary material for: The Antiquity of the Rhine River: Stratigraphic Coverage of the Dinotheriensande (Eppelsheim Formation) of the Mainz Basin (Germany)
Source: PLoS One. 2012 May 16;7(5):e36817. doi: 10.1371/journal.pone.0036817 (PMC3353959; doi:10.1371/journal.pone.0036817)
Supplement: Table S1 — Metric data of upper and lower P3-M1 molars of Deinotherium species as used in Fig. 4 of the manuscript. (DOCX) [file pone.0036817.s001.docx]

| **P3** | | | |
| --- | --- | --- | --- |
| **Locality** | **Coll. Nr./Reference** | **Lmax** | **Bmax** |
| Sprendlingen 2 | SSN12SP4 | 55,2 | 52,7 |
| Sprendlingen 2 | SSN12SP5 | 55,8 | 49,8 |
| Sprendlingen 2 | SSN12SP6 | 56 | 54 |
| Sprendlingen 2 | SSN12SP7 | 49,9 | 47,1 |
| Sprendlingen 2 | P3689 | 55,8 | 50 |
|  | | | |
| ***Deinotherium bavaricum***  **MN 5** | | **Lmax** | **Bmax** |
| Falun d'Anjou/Touraine_Noyant/Lude | Ginsburg & Chevrier 2001 | 50,2 | 47,1 |
| Falun d'Anjou/Touraine_Noyant/Lude | Ginsburg & Chevrier 2001 | 55,7 | 55 |
| Falun d'Anjou/Touraine_Noyant/Lude | Ginsburg & Chevrier 2001 | 56,1 | 50,9 |
| Falun d'Anjou/Touraine_Noyant/Lude | Ginsburg & Chevrier 2001 | 57,2 | 60 |
| Falun d'Anjou/Touraine_Noyant/Lude | Ginsburg & Chevrier 2001 | 51,3 | 50 |
| Falun d'Anjou/Touraine_Meon | Ginsburg & Chevrier 2001 | 52 | 51,6 |
| Falun d'Anjou/Touraine_Breil | Ginsburg & Chevrier 2001 | 51,3 | 51,2 |
| Falun d'Anjou/Touraine_Meigne/Vicompte | Ginsburg & Chevrier 2001 | 44,2 | 44,3 |
| Falun d'Anjou/Touraine_Savigné/Lathan | Ginsburg & Chevrier 2001 | 53 | 49,7 |
| Falun d'Anjou/Touraine_Savigné/Lathan | Ginsburg & Chevrier 2001 | 56,5 | 58,1 |
| Falun d'Anjou/Touraine_Hommes | Ginsburg & Chevrier 2001 | 47,1 | 47,4 |
| Falun d'Anjou/Touraine_Hommes | Ginsburg & Chevrier 2001 | 49,8 | 50,5 |
| Falun d'Anjou/Touraine_Hommes | Ginsburg & Chevrier 2001 | 53,3 | 52,2 |
| Falun d'Anjou/Touraine_Hommes | Ginsburg & Chevrier 2001 | 52,7 | 50,6 |
| Falun d'Anjou/Touraine_Cléré_les_Pins | Ginsburg & Chevrier 2001 | 52,2 | 45,5 |
| Falun d'Anjou/Touraine_Cléré_les_Pins | Ginsburg & Chevrier 2001 | 52 | 49,3 |
| Falun d'Anjou/Touraine_Cléré_les_Pins | Ginsburg & Chevrier 2001 | 53,9 | 49,6 |
| Falun d'Anjou/Touraine_Pont-Boutard à Saint-Michel | Ginsburg & Chevrier 2001 | 46,9 | 45,1 |
| Falun d'Anjou/Touraine_Paulmy | Ginsburg & Chevrier 2001 | 53,8 | 55,4 |
|  | | | |
| ***Deinotherium levius* & *giganteum***  **MN 8+9** | | **Lmax** | **Bmax** |
| Massenhausen | Gräf 1957 | 71,1 | 75,4 |
| Massenhausen | Gräf 1957 | 72,2 | 73,2 |
| Hinterauerbach | Gräf 1957 | 75,7 | 74,4 |
| Hinterauerbach | Gräf 1957 | 74 | 75,6 |
| Massenhausen | Gräf 1957 | 72,3 | 73,1 |
| Schalchen | Thenius 1952 | 74,3 | 71,3 |
| Oberhollabrunn | Thenius 1952 | 71,3 | 69,2 |
| Eichkogel | Thenius 1952 | 73 | 72,9 |
| Gaiselberg | Zapfe 1949 | 73 | 66 |
| Atzelsdorf | Göhlich & Huttunen 2009 | 67 | 65 |
| Atzelsdorf | Göhlich & Huttunen 2009 | 75 | 71 |
| Eppelsheim | Gräf 1957 | 74,9 | 73,4 |
|  | | | |
| ***Deinotherium giganteum***  **MN 10** | | **Lmax** | **Bmax** |
| Montredon | Tobien 1988 | 83,5 | 79,4 |
| Montredon | Tobien 1988 | 82,3 | 82,3 |
| Montredon | Tobien 1988 | 82,8 | 80,1 |
| Montredon | Tobien 1988 | 82 | 80,5 |
| Montredon | Tobien 1988 | 80 | 83 |
| Montredon | Tobien 1988 | 78 | 74,5 |
| Montredon | Tobien 1988 | 77,8 | 80,9 |
| Montredon | Tobien 1988 | 76,5 | 75,8 |
| Montredon | Tobien 1988 | 76,1 | 74,5 |
| Montredon | Tobien 1988 | 75,4 | 76,2 |
| Montredon | Tobien 1988 | 75,3 | 73,7 |
| Montredon | Tobien 1988 | 75 | 74 |
| Montredon | Tobien 1988 | 74 | 72,5 |
| Montredon | Tobien 1988 | 73,1 | 74,7 |
| Montredon | Tobien 1988 | 72,4 | 71 |
| Montredon | Tobien 1988 | 71,1 | 72,8 |
| Montredon | Tobien 1988 | 71,4 | 77 |
| Montredon | Tobien 1988 | 68,4 | 77,7 |
| Montredon | Tobien 1988 | 63,6 | 69,8 |

| **P4** | | | |
| --- | --- | --- | --- |
| **Locality** | **Coll. Nr./Reference** | **Lmax** | **Bmax** |
| Sprendlingen 2 | SSN12SP8 | 53,1 | 57 |
| Sprendlingen 2 | SSN12SP9 | 56,1 | 63,1 |
| Sprendlingen 2 | SSN12SP10 | 54,3 | 58,8 |
| Sprendlingen 2 | SSN12SP11 | 53,5 | 54,9 |
| Sprendlingen 2 | P3872 | 57,2 | 58,6 |
| Sprendlingen 2 | P3865 | 54,3 | 60,8 |
| Sprendlingen 2 | P3866 | 51,6 | 55,9 |
| Sprendlingen 2 | P3700 | 53,4 | 57 |
| Sprendlingen 2 | P3690 | 56 | 61 |
|  | | | |
| ***Deinotherium bavaricum***  **MN 5** | | **Lmax** | **Bmax** |
| Falun d'Anjou/Touraine_Pontigne | Ginsburg & Chevrier 2001 | 58 | 61 |
| Falun d'Anjou/Touraine_Noyant/Lude | Ginsburg & Chevrier 2001 | 46,3 | 53,4 |
| Falun d'Anjou/Touraine_Noyant/Lude | Ginsburg & Chevrier 2001 | 55,5 | 59,6 |
| Falun d'Anjou/Touraine_Noyant/Lude | Ginsburg & Chevrier 2001 | 47,4 | 48,5 |
| Falun d'Anjou/Touraine_Noyant/Lude | Ginsburg & Chevrier 2001 | 51,7 | 54,4 |
| Falun d'Anjou/Touraine_Noyant/Lude | Ginsburg & Chevrier 2001 | 60,9 | 49,4 |
| Falun d'Anjou/Touraine_Noyant/Lude | Ginsburg & Chevrier 2001 | 55 | 59,4 |
| Falun d'Anjou/Touraine_Meigne/Vicompte | Ginsburg & Chevrier 2001 | 48,2 | 52,4 |
| Falun d'Anjou/Touraine_Channay/Lathan | Ginsburg & Chevrier 2001 | 50,1 | 53,7 |
| Falun d'Anjou/Touraine_Savigné/Lathan | Ginsburg & Chevrier 2001 | 56,1 | 57,4 |
| Falun d'Anjou/Touraine_Savigné/Lathan | Ginsburg & Chevrier 2001 | 46,3 | 52 |
| Falun d'Anjou/Touraine_Savigné/Lathan | Ginsburg & Chevrier 2001 | 52,8 | 56,3 |
| Falun d'Anjou/Touraine_Savigné/Lathan | Ginsburg & Chevrier 2001 | 46,8 | 49 |
| Falun d'Anjou/Touraine_Hommes | Ginsburg & Chevrier 2001 | 57,5 | 57,4 |
| Falun d'Anjou/Touraine_Cléré_les_Pins | Ginsburg & Chevrier 2001 | 50,3 | 58,3 |
| Falun d'Anjou/Touraine_Cléré_les_Pins | Ginsburg & Chevrier 2001 | 47,7 | 51,6 |
| Falun d'Anjou/Touraine_Manthelan | Ginsburg & Chevrier 2001 | 53,6 | 60 |
| Falun d'Anjou/Touraine_Bossée-Manthelan | Ginsburg & Chevrier 2001 | 50 | 52 |
| Falun d'Anjou/Touraine_Paulmy | Ginsburg & Chevrier 2001 | 52,3 | 59 |
|  | | | |
| ***Deinotherium levius* & *giganteum***  **MN 8+9** | | **Lmax** | **Bmax** |
| Tournan | Ginsburg & Chevrier 2001 | 63 | 68 |
| St. Gaudens | Ginsburg & Chevrier 2001 | 70 | 73,2 |
| Gaiselberg | Zapfe 1949 | 68 | 72 |
| Eppelsheim | Gräf 1957 | 64,5 | 78,8 |
| Massenhausen | Gräf 1957 | 67,2 | 76,9 |
| Hinterauerbach | Gräf 1957 | 68,8 | 76,6 |
| Hinterauerbach | Gräf 1957 | 69,9 | 78,8 |
|  | | | |
| ***Deinotherium giganteum***  **MN 10** | | **Lmax** | **Bmax** |
| Montredon | Ginsburg & Chevrier 2001 | 69,1 | 79 |
| Montredon | Ginsburg & Chevrier 2001 | 74,1 | 82,7 |
| Montredon | Tobien 1988 | 78 | 83,5 |
| Montredon | Tobien 1988 | 77 | 88 |
| Montredon | Tobien 1988 | 76,7 | 85 |
| Montredon | Tobien 1988 | 75 | 90,2 |
| Montredon | Tobien 1988 | 75 | 82,4 |
| Montredon | Tobien 1988 | 74,4 | 87,7 |
| Montredon | Tobien 1988 | 74,2 | 81 |
| Montredon | Tobien 1988 | 74 | 81,7 |
| Montredon | Tobien 1988 | 71,8 | 82,2 |
| Montredon | Tobien 1988 | 71 | 82,2 |
| Montredon | Tobien 1988 | 70 | 78 |
| Montredon | Tobien 1988 | 69,5 | 81,5 |
| Montredon | Tobien 1988 | 69 | 78,1 |
| Montredon | Tobien 1988 | 68,5 | 76,3 |
| Montredon | Tobien 1988 | 67,1 | 75 |
| Montredon | Tobien 1988 | 67,1 | 79,4 |
| Montredon | Tobien 1988 | 67 | 78 |
| Montredon | Tobien 1988 | 76,1 | 78,1 |
| Montredon | Tobien 1988 | 63,1 | 71 |

| **M1** | | | |
| --- | --- | --- | --- |
| **Locality** | **Coll. Nr./Reference** | **Lmax** | **Bmax** |
| Sprendlingen 2 | SSN12SP12 | 88,6 | 73 |
| Sprendlingen 2 | SSN12SP13 | 68,3 | 55 |
| Sprendlingen 2 | SSN12SP14 | 71,2 | 60 |
| Sprendlingen 2 | SSN12SP15 | 90,2 | 74,1 |
| Sprendlingen 2 | SSN12SP16 | 72 | 61,2 |
|  | | | |
| ***Deinotherium bavaricum***  **MN 5** | | **Lmax** | **Bmax** |
| Falun d'Anjou/Touraine_Genneteil | Ginsburg & Chevrier 2001 | 72,5 | 58,1 |
| Falun d'Anjou/Touraine_Noyant/Lude | Ginsburg & Chevrier 2001 | 66,5 | 56,6 |
| Falun d'Anjou/Touraine_Noyant/Lude | Ginsburg & Chevrier 2001 | 71,3 | 57,2 |
| Falun d'Anjou/Touraine_Noyant/Lude | Ginsburg & Chevrier 2001 | 72 | 61 |
| Falun d'Anjou/Touraine_Noyant/Lude | Ginsburg & Chevrier 2001 | 73,6 | 54,5 |
| Falun d'Anjou/Touraine_Meon | Ginsburg & Chevrier 2001 | 66,1 | 53,8 |
| Falun d'Anjou/Touraine_Channay/Lathan | Ginsburg & Chevrier 2001 | 70,4 | 57,2 |
| Falun d'Anjou/Touraine_Savigné/Lathan | Ginsburg & Chevrier 2001 | 71,4 | 60,8 |
| Falun d'Anjou/Touraine_Savigné/Lathan | Ginsburg & Chevrier 2001 | 67,8 | 54,9 |
| Falun d'Anjou/Touraine_Savigné/Lathan | Ginsburg & Chevrier 2001 | 70,8 | 61,8 |
| Falun d'Anjou/Touraine_Hommes | Ginsburg & Chevrier 2001 | 70,8 | 58 |
| Falun d'Anjou/Touraine_Hommes | Ginsburg & Chevrier 2001 | 67,9 | 55,8 |
| Falun d'Anjou/Touraine_Hommes | Ginsburg & Chevrier 2001 | 69,5 | 58,6 |
| Falun d'Anjou/Touraine_Cléré_les_Pins | Ginsburg & Chevrier 2001 | 72,5 | 58 |
| Falun d'Anjou/Touraine_Cléré_les_Pins | Ginsburg & Chevrier 2001 | 73,1 | 60,1 |
| Falun d'Anjou/Touraine_Bossée | Ginsburg & Chevrier 2001 | 68,8 | 62,6 |
| Falun d'Anjou/Touraine_Bossée | Ginsburg & Chevrier 2001 | 67,8 | 56,5 |
|  | | | |
| ***Deinotherium levius* & *giganteum***  **MN 8+9** | | **Lmax** | **Bmax** |
| Tournan | Ginsburg & Chevrier 2001 | 81,6 | 71,6 |
| Massenhausen | Gräf 1957 | 84,4 | 75 |
| Env. Vienne-en-Dauphine | Ginsburg & Chevrier 2001 | 99,5 | 83,1 |
| Env. Vienne-en-Dauphine | Ginsburg & Chevrier 2001 | 94,5 | 81,3 |
| Eppelsheim | Gräf 1957 | 91,7 | 77,7 |
| Eppelsheim | Gräf 1957 | 92,9 | 77,6 |
| Massenhausen | Gräf 1957 | 84,7 | 74,4 |
| Massenhausen | Gräf 1957 | 88,3 | 71,5 |
| Hinterauerbach | Gräf 1957 | 88,6 | 77,9 |
|  | | | |
| ***Deinotherium giganteum***  **MN 10** | | **Lmax** | **Bmax** |
| Montredon | Ginsburg & Chevrier 2001 | 94,4 | 78 |
| Montredon | Tobien 1988 | 105,5 | 85 |
| Montredon | Tobien 1988 | 103 | 84,7 |
| Montredon | Tobien 1988 | 95,5 | 83,7 |
| Montredon | Tobien 1988 | 90 | 82 |
| Montredon | Tobien 1988 | 90 | 72,1 |
| Montredon | Tobien 1988 | 87,7 | 71,5 |
| Montredon | Tobien 1988 | 88,6 | 73,6 |
| Montredon | Tobien 1988 | 88 | 73 |
| Montredon | Tobien 1988 | 88 | 78,4 |

| **p3** | | | |
| --- | --- | --- | --- |
| **Locality** | **Coll. Nr./Reference** | **Lmax** | **Bmax** |
| Sprendlingen 2 | SSN12SP28 | 42,7 | 34,5 |
| Sprendlingen 2 | SSN12SP29 | 47 | 36,3 |
| Sprendlingen 2 | SSN12SP30 | 47,2 | 35,4 |
| Sprendlingen 2 | SSN12SP31 | 65,5 | 54,3 |
| Sprendlingen 2 | SSN12SP32 | 54,9 | 42,8 |
| Sprendlingen 2 | SSN12SP33 | 58 | 54,1 |
| Sprendlingen 2 | P3867 | 47,6 | 40 |
| Sprendlingen 2 | P3869 | 45,7 | 35 |
|  | | | |
| ***Deinotherium bavaricum***  **MN 5** | | **Lmax** | **Bmax** |
| Falun d'Anjou/Touraine_Auverse | Ginsburg & Chevrier 2001 | 42,8 | 35,5 |
| Falun d'Anjou/Touraine_Noyant/Lude | Ginsburg & Chevrier 2001 | 46 | 35,3 |
| Falun d'Anjou/Touraine_Noyant/Lude | Ginsburg & Chevrier 2001 | 45,1 | 34,4 |
| Falun d'Anjou/Touraine_Noyant/Lude | Ginsburg & Chevrier 2001 | 41,2 | 29,6 |
| Falun d'Anjou/Touraine_Noyant/Lude | Ginsburg & Chevrier 2001 | 43,5 | 35,9 |
| Falun d'Anjou/Touraine_Meigne/Vicompte | Ginsburg & Chevrier 2001 | 45,8 | 36,9 |
| Falun d'Anjou/Touraine_Channay/Lathan | Ginsburg & Chevrier 2001 | 44,2 | 36,8 |
| Falun d'Anjou/Touraine_Channay/Lathan | Ginsburg & Chevrier 2001 | 40,4 | 32,6 |
| Falun d'Anjou/Touraine_Savigné/Lathan | Ginsburg & Chevrier 2001 | 41,4 | 34,3 |
| Falun d'Anjou/Touraine_Savigné/Lathan | Ginsburg & Chevrier 2001 | 37,2 | 29,3 |
| Falun d'Anjou/Touraine_Hommes | Ginsburg & Chevrier 2001 | 41 | 26,8 |
| Falun d'Anjou/Touraine_Cléré_les_Pins | Ginsburg & Chevrier 2001 | 48,4 | 37,6 |
| Falun d'Anjou/Touraine_Cléré_les_Pins | Ginsburg & Chevrier 2001 | 51 | 37,8 |
| Falun d'Anjou/Touraine_Bossée-Manthelan | Ginsburg & Chevrier 2001 | 44 | 33 |
|  | | | |
| ***Deinotherium levius* & *giganteum***  **MN 8+9** | | **Lmax** | **Bmax** |
| Massenhausen | Gräf 1957 | 57,9 | 48,1 |
| Hinterauerbach | Gräf 1957 | 60 | 48,3 |
| Hinterauerbach | Gräf 1957 | 58,2 | 51,9 |
| Türkenschanze | Huttunen 2002 | 59 | 39 |
| Kettlasbrunn | Bachmayer & Zapfe 1976 | 68 | 56 |
| Langon | Ginsburg & Chevrier 2001 | 73 | 57,5 |
| Frohnstetten | Gräf 1957 | 66,9 | 50,6 |
| Frohnstetten | Gräf 1957 | 61,8 | 52,3 |
| Eppelsheim | Gräf 1957 | 67 | 47,5 |
| Eppelsheim | Gräf 1957 | 60,9 | 49,2 |
|  | | | |
| ***Deinotherium giganteum***  **MN 10** | | **Lmax** | **Bmax** |
| Montredon | Tobien 1988 | 70,1 | 54,1 |
| Montredon | Tobien 1988 | 66,2 | 55,4 |
| Montredon | Tobien 1988 | 66,1 | 60 |
| Montredon | Tobien 1988 | 65,1 | 52,5 |
| Montredon | Tobien 1988 | 63,8 | 51,6 |
| Montredon | Tobien 1988 | 65 | 52 |
| Montredon | Tobien 1988 | 65 | 50 |
| Montredon | Tobien 1988 | 65 | 50,5 |
| Montredon | Tobien 1988 | 64,5 | 55,6 |
| Montredon | Tobien 1988 | 60,9 | 53 |
| Montredon | Tobien 1988 | 58 | 52,1 |

| **p4** | | | |
| --- | --- | --- | --- |
| **Locality** | **Coll. Nr./Reference** | **Lmax** | **Bmax** |
| Sprendlingen 2 | SSN12SP34 | 66,8 | 57,3 |
| Sprendlingen 2 | P3742 | 60 | 51,1 |
| Sprendlingen 2 | P3870 | 58 | 46,2 |
| Sprendlingen 2 | P3699 | 70 | 58,9 |
|  | | | |
| ***Deinotherium bavaricum***  **MN 5** | | **Lmax** | **Bmax** |
| Falun d'Anjou/Touraine_Noyant/Lude | Ginsburg & Chevrier 2001 | 52,5 | 44,8 |
| Falun d'Anjou/Touraine_Noyant/Lude | Ginsburg & Chevrier 2001 | 60 | 49,9 |
| Falun d'Anjou/Touraine_Noyant/Lude | Ginsburg & Chevrier 2001 | 53,3 | 44,5 |
| Falun d'Anjou/Touraine_Noyant/Lude | Ginsburg & Chevrier 2001 | 54,9 | 47 |
| Falun d'Anjou/Touraine_Breil | Ginsburg & Chevrier 2001 | 50,8 | 44,4 |
| Falun d'Anjou/Touraine_Meigne/Vicompte | Ginsburg & Chevrier 2001 | 60 | 51 |
| Falun d'Anjou/Touraine_Savigné/Lathan | Ginsburg & Chevrier 2001 | 43,8 | 42,8 |
| Falun d'Anjou/Touraine_Savigné/Lathan | Ginsburg & Chevrier 2001 | 59,8 | 53,2 |
| Falun d'Anjou/Touraine_Savigné/Lathan | Ginsburg & Chevrier 2001 | 51,2 | 42,7 |
| Falun d'Anjou/Touraine_Savigné/Lathan | Ginsburg & Chevrier 2001 | 46,6 | 37,5 |
| Falun d'Anjou/Touraine_Hommes | Ginsburg & Chevrier 2001 | 53,9 | 43,7 |
| Falun d'Anjou/Touraine_Hommes | Ginsburg & Chevrier 2001 | 52,8 | 44 |
| Falun d'Anjou/Touraine_Cléré_les_Pins | Ginsburg & Chevrier 2001 | 53,6 | 46,2 |
| Falun d'Anjou/Touraine_Cléré_les_Pins | Ginsburg & Chevrier 2001 | 56,6 | 44 |
| Falun d'Anjou/Touraine_Cléré_les_Pins | Ginsburg & Chevrier 2001 | 53,6 | 44 |
| Falun d'Anjou/Touraine_Cléré_les_Pins | Ginsburg & Chevrier 2001 | 52 | 44,3 |
| Falun d'Anjou/Touraine_Pont-Boutard à Saint-Michel | Ginsburg & Chevrier 2001 | 56,1 | 45,4 |
| Falun d'Anjou/Touraine_Pont-Boutard à Saint-Michel | Ginsburg & Chevrier 2001 | 38 | 38 |
|  | | | |
| ***Deinotherium levius* & *giganteum***  **MN 8+9** | | **Lmax** | **Bmax** |
| Massenhausen | Gräf 1957 | 67 | 58,2 |
| Massenhausen | Gräf 1957 | 70,2 | 58,9 |
| Hinterauerbach | Gräf 1957 | 69,1 | 59,6 |
| Hinterauerbach | Gräf 1957 | 68,1 | 60,6 |
| Tournan | Ginsburg & Chevrier 2001 | 66 | 57,4 |
| Frohnstetten | Gräf 1957 | 72,9 | 60,7 |
| Frohnstetten | Gräf 1957 | 70,2 | 61,5 |
| Eppelsheim | Gräf 1957 | 73,7 | 59,8 |
| Eppelsheim | Gräf 1957 | 65,2 | 54,9 |
| Belvedere Wien 3 | Huttunen 2002 | 77 | 57 |
| Oswaldgasse Wien 12 | Huttunen 2002 | 63 | 55 |
| Hollabrunn | Huttunen 2002 | 67 | 48 |
| Sauerbrunn | Steininger & Thenius 1965 | 64 | 55 |
| Mistelbach | Steininger & Thenius 1965 | 60,5 | 56 |
| Kettlasbrunn | Bachmayer & Zapfe 1976 | 73 | 63,5 |
|  | | | |
| ***Deinotherium giganteum***  **MN 10** | | **Lmax** | **Bmax** |
| Montredon | Tobien 1988 | 80,3 | 65,8 |
| Montredon | Tobien 1988 | 79,1 | 66,5 |
| Montredon | Tobien 1988 | 79,1 | 62,5 |
| Montredon | Tobien 1988 | 76,7 | 60,6 |
| Montredon | Tobien 1988 | 76,1 | 63,5 |
| Montredon | Tobien 1988 | 75 | 60,5 |
| Montredon | Tobien 1988 | 75 | 63,4 |
| Montredon | Tobien 1988 | 74,3 | 61 |
| Montredon | Tobien 1988 | 73 | 61,7 |
| Montredon | Tobien 1988 | 72 | 58,1 |
| Montredon | Tobien 1988 | 68,5 | 56,6 |
| Montredon | Tobien 1988 | 68,2 | 55,6 |
| Montredon | Tobien 1988 | 68,2 | 58 |

| **m1** | | | |
| --- | --- | --- | --- |
| **Locality** | **Coll. Nr./Reference** | **Lmax** | **Bmax** |
| Sprendlingen 2 | P3863 | 71,9 | 50 |
| Sprendlingen 2 | P3895 | 66,5 | 44,3 |
| Sprendlingen 2 | P3736 | 65,2 | 44,8 |
|  | | | |
| ***Deinotherium bavaricum***  **MN 5** | | **Lmax** | **Bmax** |
| Falund d'Anjou/Touraine_Pontigne | Ginsburg & Chevrier 2001 | 71,5 | 49 |
| Falun d'Anjou/Touraine_Noyant/Lude | Ginsburg & Chevrier 2001 | 61,5 | 47 |
| Falun d'Anjou/Touraine_Noyant/Lude | Ginsburg & Chevrier 2001 | 66 | 46 |
| Falun d'Anjou/Touraine_Noyant/Lude | Ginsburg & Chevrier 2001 | 60 | 41,7 |
| Falun d'Anjou/Touraine_Breil | Ginsburg & Chevrier 2001 | 73,1 | 47,8 |
| Falun d'Anjou/Touraine_Deneze | Ginsburg & Chevrier 2001 | 69,2 | 45,2 |
| Falun d'Anjou/Touraine_Savigné/Lathan | Ginsburg & Chevrier 2001 | 69 | 47 |
| Falun d'Anjou/Touraine_Hommes | Ginsburg & Chevrier 2001 | 70,3 | 48,5 |
| Falun d'Anjou/Touraine_Cléré_les_Pins | Ginsburg & Chevrier 2001 | 68 | 45 |
| Falun d'Anjou/Touraine_Pont-Boutard à Saint-Michel | Ginsburg & Chevrier 2001 | 52,5 | 39,5 |
|  | | | |
| ***Deinotherium levius* & *giganteum***  **MN 8+9** | | **Lmax** | **Bmax** |
| Massenhausen | Gräf 1957 | 83,7 | 59,7 |
| Hinterauerbach | Gräf 1957 | 85,7 | 62,3 |
| Hinterauerbach | Gräf 1957 | 82,8 | 60,3 |
| Cassagnabere | Ginsburg & Chevrier 2001 | 78,3 | 69,2 |
| Montesquieu-Guittaut | Ginsburg & Chevrier 2001 | 92,6 | 60,4 |
| Kettlasbrunn | Bachmayer & Zapfe 1976 | 81 | 59 |
| Belvedere Wien 3 | Huttunen 2002 | 88 | 58 |
| Belvedere Wien 4 | Huttunen 2002 | 84 | 58 |
| Eppelsheim | Gräf 1957 | 96,4 | 64,5 |
| Eppelsheim | Gräf 1957 | 95,3 | 58,8 |
|  | | | |
| ***Deinotherium giganteum***  **MN 10** | | **Lmax** | **Bmax** |
| Montredon | Tobien 1988 | 95 | 65 |
| Montredon | Tobien 1988 | 93,1 | 63,8 |
| Montredon | Tobien 1988 | 90 | 59,6 |
| Montredon | Tobien 1988 | 90 | 60 |
| Montredon | Tobien 1988 | 90 | 64 |

**Reference**

Bachmayer, F., Zapfe, H. (1976) Ein bedeutender Fund von Dinotherium aus dem Pannon von Niederösterreich. Annalen des Naturhistorischen Museum Wien 80: 145-162.

Ginsburg, L., Chevrier, F. (2001). Les Dinothères du bassin de la Loire et l`évolution du genre *Deinotherium* en France. Symbioses 5: 9-24.

Göhlich, U.B., Huttunen, K. (2009). The early Vallesian vertebrates of Atzelsdorf (Austria, Late Miocene). 12. Proboscidea. Annalen des Naturhistorischen Museum Wien 111A: 635-646.

Gräf, I.E. (1957). Die Prinzipien der Artbestimmung bei *Dinotherium*. Palaeontographica A 108: 131-185.

Huttunen, K. (2002). Deinotheriidae (Proboscidea, Mammalia) dental remains from the Miocene of Lower Austria and Burgenland. Annalen des Naturhistorischen Museum Wien A 103: 251-285.

Steininger, F., Thenius, E. (1965). Eine Wirbeltierfauna aus dem Sarmat (Ober-Miozän) von Sauerbrunn (Burgenland). – Mitteilungen der Geologischen Gesellschaft Wien 7: 449-467.

Tobien H (1988) Contributions a l’étude du gisement miocène supérieur de Montredon (Herault). Les grands Mammifères. 7 - Les proboscidiens Deinotheriidae. Palaeovertebrata, Mémoire Extraordinaire: 135-175.

Zapfe, H. (1949). Die Säugetierfauna aus dem Unterpliozän von Gaiselberg bei Zistersdorf in Niederösterreich. Jahrbuch der geologischen Bundesanstalt 93: 83-97.
