# Supplementary material for: The Antiquity of the Rhine River: Stratigraphic Coverage of the Dinotheriensande (Eppelsheim Formation) of the Mainz Basin (Germany)
Source: PLoS One. 2012 May 16;7(5):e36817. doi: 10.1371/journal.pone.0036817 (PMC3353959; doi:10.1371/journal.pone.0036817)
Supplement: Table S2 — List of cervid localities from Central Europe and the reference regarding taxonomy and/or biostratigraphy. (DOCX) [file pone.0036817.s002.docx]

| Locality | Reference | Locality | Reference |
| --- | --- | --- | --- |
| Wintershof-West | Obergfell 1957, Rössner 1995, 1997 | Haberskirch | Dehm 1957 |
| Petersbuch 2 | Rössner 1997 | Göriach | Thenius 1950 |
| Erkertshofen 2 | Rössner 1997 | Kirrberg | Seehuber 2008 |
| Oberdorf | Rössner 1998 | Klein-Hadersdorf | Thenius 1948b |
| Baltringen | Rütimeyer 1881, Dehm 1951 | Friedberg/Stmk. | Thenius 1950 |
| Eggingen-Mittelhart 3 | Sach & Heizmann 2001 | Mikulov (=Nikolsburg) | Buday et al. 1964; Thenius 1948b, 1959 |
| Langenau | Sach & Heizmann 2001 | Derndorf | Seehuber 2008 |
| Jungnau | Dehm 1951 | Devinska Nova Ves Fissure | Thenius 1959 |
| Rauscheröd | Rössner 1995 | Steinheim | Stehlin 1928 |
| Gerlenhofen | Sach & Heizmann 2001 | Eppishausen | Seehuber 2008 |
| Franzensbad | Fejfar & Kvacek 1993 | Devinska Nova Ves Sandberg | Thenius 1952 |
| Reisensburg | Dehm 1944 | Breitenbrunn | Seehuber 2008 |
| Langenmoosen | Dehm 1952 | Mörgen | Seehuber 2008 |
| Heggbach | Rütimeyer 1881 | Anwil | Engesser 1972 |
| Sandelzhausen | Rössner 2011 | Türkenschanze | Thenius 1948b |
| Häder | Roger 1904, Dehm 1944, 1957 | Heiligenstadt | Thenius 1948b |
| Schwanberg | Thenius 1950, Mottl 1970, van der Made 2010 | Massenhausen | Dehm in Jung 1963 |
| Seegraben | Thenius 1950; van der Made 2010 | Przeworno | Glazek et al. 1971 |
| Brüchl | New data | Gratkorn | Gross et al. 2011 |
| Deisenhausen | New data | Atzgersdorf | Thenius 1948b |
| Burg-Balzhausen | Seehuber 2008 | Aumeister | Stromer 1940 |
| Hambach 6C | Mörs et al. 2000 | Atzelsdorf | Hillenbrandt et al. 2009 |
| Feisternitz | Mottl 1979, van der Made 2010 | Mariathal | Thenius 1982 |
| Griesbeckerzell 1a | Kaiser & Rössner 2007 | Ilz | Thenius 1950, Mottl 1970 |
| Ziemetshausen 1b | Heissig 1989 | Wien III | Thenius 1948b |
| Derching | Seehuber 2002 | Laaerberg | Thenius 1948b |
| Pfaffenzell 1 | Seehuber 2002; Eronen & Rössner 2007 | Vösendorf | Thenius 1948a, 1966; Papp & Thenius 1954 |
| Gallenbach 2b | Heissig 1989 | Tataros | Rabeder 1985 |
| Gallenbach 3a | Heissig 1989 | Rudabanya | Bernor et al. 2004 |
| Wies | Thenius 1950 | Mönchhof | Zapfe 1997 |
| Thannhausen | Kaiser & Rössner 2007 | Himberg | Thenius 1948b |
| Stätzling | Roger 1900, Dehm 1957 | Nikitsch | Thenius 1956; Vislobokova 2006 |
| Laimering 3a | Rössner 2006 |  |  |

**Reference**

Bernor, R.L., Kordos, L., Rook, L., Agustí, J., Andrews, P., Armour-Chelu, M., Begun, D.R., Cameron, D.W., Damuth, J., Daxner-Höck, G., De Bonis, L., Fejfar, O., Fessaha, N., Fortelius, M., Franzen, J., Gasparik, M., Gentry, A., Heissig, K., Hernyak, G., Kaiser, T., Koufos, G.D., Krolopp, E., Jánossy, D., Llenas, M., Meszáros, L., Müller, P., Renne, P., Roček, Z., Sen, S., Scott, R., Szyndlar, Z., Topál, G., Ungar, P.S., Utescher, T., Van Dam, J.A., Werdelin, L. & Ziegler, R. (2004). Recent Advances on Multidisciplinary Research at Rudabánya, Late Miocene (MN9), Hungary: a compendium. Paleontographia Italica 89: 18.

Buday, T., Cicha, I., Ctyroky, P., Fejfar, O. (1964). Die Stellung des Neogens der Westkarpaten in der Paratethys. Cursillos y Conferencias del Instituto Lucas Mallada IX (1964): 109-116.

Dehm, R. (1944). Frühe Hirschgeweihe aus dem Miocän Süddeutschlands. Neues Jahrbuch für Mineralogie, Geologie und Paläontologie, Monatshefte 1944 B(4): 81–98.

Dehm, R. (1951). Zur Gliederung der jungtertiären Molasse in Süddeutschland nach Säugetieren. Neues Jahrbuch für Geologie und Palä ontologie, Monatshefte: 140–152.

Dehm, R. (1952). Über den Fossilinhalt von Aufarbeitungslagen im tieferen Ober-Miocän Südbayerns. Geologica Bavarica 14: 86-90.

Dehm, R. (1957). Fossilführung und Altersbestimmung der Oberen Süßwasser-Molasse auf Blatt Augsburg 1: 50.000. Erläuterungen geologische Karte Augsburg, p. 34-39; Bayerisches Geologisches Landesamt, München.

Engesser B. (1972). Die obermiozäne Säugertierefauna von Anwil (Baselland). Tätigkeitsberichte der Naturforschenden Gesellschaft Baselland 28: 37-363.

Eronen, J.T., Rössner, G.E. (2007). Wetland paradise lost: Miocene community dynamics in large herbivorous mammals from the German Molasse Basin. In: Evolutionary Ecology Research 9: 471-494.

Fejfar, O., Kvacek, Z. (1993). Tertiary Basins in Northwest Bohemia – Excursion Nr. 3. Excursion Guide, 63. Jahrestagung Paläontologische Gesellschaft, 21-26. September 1993, 35 p.; Universita Karlova, Ceska geologicka spolecnost.

Glazek, J., Oberc, J., Sulimski, A. (1971). Miocene vertebrate faunas from Przeworno (Lower Silesia) and their geological setting. Acta Geologica Polonica 21 (3): 473-516.

Gross, M., Böhme, M., Prieto, J. (2011). Gratkorn: A benchmark locality for the continental Sarmatian s.str. of the Central Paratethys. International Journal of Earth Sciences (Geologische Rundschau) 100 (8): 1895-1913.

Kaiser, T., Rössner, G.E. (2007). Dietary resource partitioning in ruminant communities of Miocene wetland and karst palaeoenvironments in Southern Germany. Palaeogeography, Palaeoclimatology, Palaeoecology **252** (3-4): 424-439.

Heissig, K. (1989) Neue Ergebnisse zur Statigraphie der Mittleren Serie der Oberen Süßwassermolasse Bayerns. Geologica Bavarica 94: 239–258.

Hillenbrand, V., Göhlich, U.B., Rössner, G.E. (2009). The early Vallesian vertebrates of Atzelsdorf (Late Miocene, Austria) 7. Ruminantia. Annalen des Naturhistorischen Museums in Wien 111A: 519-556.

Jung, W. (1963): Blatt- und Fruchtreste aus der Oberen Süßwassermolasse von Massenhausen, Kreis Freising (Oberbayern).- Palaeontographica 12B, 166 S.

Mörs, Th., von der Hocht, F., Wutzler, B., 2000. Die erste Wirbeltierfauna aus der miozänen Braunkohle der Nieder-rheinischen Bucht (Ville-Schichten, Tagebau Hambach). Paläontologische Zeitschrift 74: 145-170.

Mottl, M. (1970). Die jungtertiären Säugetierfaunen der Steiermark, Südost-Österreichs. Mitteilungen des Museums für Bergbau, Geologie und Technik am Landesmuseum Joanneum, Graz 31: 3–92.

Obergfell, F. A., 19 57. Vergleichende Untersuchungen an den Dentitionen und Dentale altburdigaler Cerviden von Wintershof-West in Bayern und rezenter Cerviden (eine phylogenetische Studie). Palaeontographica A 109 (3/6): 71–166.

Papp, A., Thenius, E. (1954). Vösendorf – ein Lebensbild aus dem Pannon des Wiener Beckens. Mitteilungen der Geologischen Gesellschaft in Wien 46, 109 p.

Rabeder, G. (1985): Die Säugetiere des Pannonien. - In: Papp, A., Jambor, A. & Steininger, F. (Eds): Chronostratigraphie und Neostratotypen. Miozän der Zentralen Paratethys. M6 Pannonien, 440-463; Budapest (Ungarischer Akademie der Wissenschaften).

Roger, O. (1900). Wirbelthierreste aus dem Dinotheriensande III. Theil. Bericht des Naturwissenschaftlichen Vereins für Schwaben und Neuburg in Augsburg 34: 55–70.

Roger, O. (1904). Wirbelthierreste aus dem Dinotheriensande der bayerisch-schwäbischen Hochebene (V. Theil). Bericht des Naturwissenschaftlichen Vereins für Schwaben und Neuburg in Augsburg 36: 3-22.

Rössner, G. E. (1995). Odontologische und schädelanatomische Untersuchungen. Münchner Geowissenschaftliche Abhandlungen A 29: 127 p.

Rössner, G.E. (1997). Biochronology of Ruminant Assemblages in the Lower Miocene of Southern Germany. In: Aguilar, J.-P.; Legendre, S. & Michaux, J. (Hrsg.): Actes du Congrès BiochroM`97. Mémoires et Travaux de l`E.P.H.E., Institut de Montpellier **21**: 609-618.

Rössner, G.E. (1998). Wirbeltiere aus dem Unter-Miozän des Lignit-Tagebaues Oberdorf (Weststeirisches Becken, Österreich): 9. Ruminantia (Mammalia). Annalen des Naturhistorischen Museums in Wien 99A: 169-193.

Rössner, G. E. (2006). A community of Middle Miocene Ruminantia (Mammalia, Artiodactyla) from the German Molasse Basin. Palaeontographica A 277(1-6): 103-112.

Rössner, G. E. (2010). Systematics and palaeoecology of the Ruminantia (Artiodactyla, Mammalia) community from Sandelzhausen (Early/Middle Miocene) in the German Molasse Basin. Paläontologische Zeitschrift 84(1): 123-162.

Rütimeyer, L. (1881). Beiträge zu einer natürlichen Geschichte der Hirsche. Erster Teil. Abhandlungen der schweizerischen paläontologischen Gesellschaft VIII: 1–93.

Sach, V., Heizmann, E.P.J. (2001). Stratigraphie und Säugetierfaunen der Brackwassermolasse in der Umgebung von Ulm (Südwestdeutschland). Stuttgarter Beiträge zur Naturkunde B 310: 1–95.

Stromer, E. (1940). Die jungtertiäre Fauna des Flinzes und des Schweiß-Sandes von München. Abhandlungen der Bayerischen Akademie der Wissenschaften, mathematisch-nasturwissenschaftliche Abteilung, N.F. 48, 102 p.

Seehuber, U. (2002). Sedimentologische und paläontologische Untersuchungen in der Oberen Süßwassermolasse östlich Derching (Landkreis Aichach-Friedberg, Bayern). Neues Jahrbuch für Geologie und Paläontologie, Abhandlungen 223: 201–239.

Seehuber, U. (2008). Litho- und biostratigraphische Untersuchungen in der Oberen Süßwassermolasse in der Umgebung von Kirchheim in Schwaben. Thesis Ludwig–Maximilians–University Munich, 276 p.

Stehlin, H. G. (1928). Bemerkungen über die Hirsche von Steinheim am Albuch. Eclogae geologicae Helvetiae 21: 245-256.

Thenius, E. (1948 a). Die Säugetierfauna aus den Congerienschichten von Brunn-Vösendorf bei Wien. Verhandlungen Geologische Bundes-Anstalt Wien 1948 (7-9): 113-131.

Thenius, E. (1948 b). Zur Kenntnis der fossilen Hirsche des Wiener Beckens, unter besonderer Berücksichtigung ihrer stratigraphischen Bedeutung. Annalen des Naturhistorischen Museums in Wien 56: 262-308.

Thenius, E. (1950). Die tertiären Lagomeryciden und Cerviden der Steiermark. Sitzungsbericht der Österreichischen Akademie der Wissenschaften, Mathematisch-naturwissenschaftlichen Klasse I 159: 219-254.

Thenius, E. (1952): Die Säugetierfauna aus dem Torton von Neudorf an der March. Neues Jahrbuh Geologie Paläontologie, Anhandlungen 96 (1): 27-136.

Thenius, E. (1956). Ein Geweih aus dem Pannon des Burgenlandes. Burgenländische Heimatblätter 18: 145-148.

Thenius, E. (1959). Wirbeltierfaunen. Tertiär 2. Teil. Handbuch der Stratigraphischen Geologie III/2, 328 p., Ferdinand Enke Verlag, Stuttgart.

Thenius, E. (1982). Ein Menschenaffenfund (Primates: Pongidae) aus dem Pannon (Jung-Miozän) von Niederösterreich. Folia Primatologica 39: 187-200.

van der Made, J. (2010). The pigs and ‘‘Old World peccaries’’ (Suidae and Palaeochoeridae, Suoidea, Artiodactyla) from the Miocene of Sandelzhausen (southern Germany): phylogeny and an updated classification of the Hyotheriinae and Palaeochoeridae. Paläontologische Zeitschrift 84(1): 43-121.

Vislobokova, I.A. (2006). Associations of Ruminants in Miocene Ecosystems of Eastern Alpine Region. Paleontological Journal 40 (4): 438–447.

Zapfe, H. (1997). Ein bemerkenswertes Cervidengeweih aus dem Pannon des Burgenlandes, Österreich. Annalen des Naturhistorischen Museums in Wien 98A: 173-177.
